# Type VI-secretion system-mediated competition and virulence in clinical and environmental *Stenotrophomonas maltophilia*

**DOI:** 10.1128/spectrum.03411-25

**Published:** 2026-02-27

**Authors:** Natália Carolina Drebes Dörr, Bárbara Bortolozzo Ribeiro, Arthur Henrique Barrios Solano, Isabella Carolina Rodrigues dos Santos Goes, Francine Coelho Rosa, Natalli Jennifer Tancredo de Oliveira, Eliana Guedes Stehling, Herrison Fontana, Marcelo Brocchi, Rodrigo S. Galhardo, João Carlos Setubal, Cristina E. Alvarez-Martinez

**Affiliations:** 1 Departamento de Genética, Evolução, Microbiologia e Imunologia, Instituto de Biologia, Universidade Estadual de Campinas 124594 , Campinas, Brazil; 2 Departamento de Bioquímica, Instituto de Química, Universidade de São Paulo 153989 , São Paulo, Brazil; 3 Departamento de Análises Clínicas, Toxicológicas e Bromatológicas, Faculdade de Ciências Farmacêuticas de Ribeirão Preto, Universidade de São Paulo, Ribeirão Preto, São Paulo, Brazil; 4 Departamento de Microbiologia, Instituto de Ciências Biomédicas, Universidade de São Paulo 525539 , São Paulo, Brazil; Universidad Maimonides, Buenos Aires, Argentina

**Keywords:** virulence, antibacterial effectors, bacterial competition

## Abstract

The escalating global threat of antimicrobial resistance highlights the urgency of understanding pathogens like *Stenotrophomonas maltophilia*, an intrinsically multidrug-resistant opportunistic bacterium. While increasingly isolated in nosocomial infections, *S. maltophilia* is also ubiquitous in diverse environmental settings. Crucially, in both clinical and environmental niches, this bacterium thrives within polymicrobial communities, making its interactions with other microorganisms particularly significant. However, the cooperative and competitive dynamics within these communities, which could be used in innovative therapeutic strategies, are largely unexplored. Our study investigated the prevalence, functional contribution, and effector diversity of the type VI secretion system (T6SS), a powerful bacterial molecular weapon, in a collection of clinical and environmental Brazilian *S. maltophilia* isolates. Through whole-genome sequencing, comparative genomics, and functional assays, we found that T6SS clusters, predominantly of the i4-clade, are present in a small but lineage-specific subset of isolates. Notably, one clinical strain also harbored an additional i3-clade T6SS. Interbacterial competition assays against *Escherichia coli* showed significant variations in killing efficacy, with a subset of isolates from the Sgn4 monophyletic lineage exhibiting robust antibacterial activity and increased pathogenicity in a *Galleria mellonella* infection model. Importantly, genetic dissection demonstrated that both phenotypes were T6SS-dependent. Further analysis of T6SS effector profiles identified diverse toxin-immunity pairs, including predicted pore-forming toxins and peptidoglycan hydrolases, correlating with observed phenotypes and suggesting possible anti-eukaryotic potential. Our results underscore the complex genomic variability and ecological strategies of the *S. maltophilia* complex (Smc), highlighting the T6SS’s importance as a mediator of competition.

**IMPORTANCE:**

*Stenotrophomonas maltophilia* is an emerging opportunistic pathogen associated with severe nosocomial infections with limited treatment options due to resistance to multiple antibiotics. Mechanisms of pathogenicity and fitness are relatively poorly understood in the species, which is ubiquitous in the environment and shows high genome plasticity. Type VI secretion systems (T6SS) are molecular machines that inject effector proteins into target cells, playing fundamental roles in bacterial interactions within polymicrobial communities and hosts. In this study, we identify and characterize T6SSs in a diverse set of clinical and environmental isolates of *S. maltophilia* from Brazil, revealing their role in bacterial antagonism and virulence. This study provides new insights into the mechanisms that contribute to *S. maltophilia*’s ability to thrive in distinct environments, also contributing to the understanding of the complex genomic variability within the species.

## INTRODUCTION

The global increase in multidrug-resistant bacteria poses a major challenge to public health, with an estimated 4.71 million deaths associated with bacterial antibiotic resistance in 2021 (1). Nosocomial infections caused by opportunistic Gram-negative bacteria are particularly difficult to treat (2), and *Stenotrophomonas maltophilia* has emerged as a significant pathogen, frequently isolated in hospitals, especially in pneumonia cases (3). *S. maltophilia* is a Gram-negative, non-fermenting, strictly aerobic bacillus. In fact, phylogenomic and taxonomic studies demonstrated that *S. maltophilia* actually comprises multiple cryptic species, leading to the adoption of the term “*S. maltophilia* complex” (Smc) to reflect this heterogeneity (4, 5). Smc includes both environmental and clinically relevant strains, the latter associated with severe nosocomial infections such as pneumonia, bacteremia, endocarditis, and meningitis (6, 7). Although considered of low virulence, Smc infections may pose a serious threat to immunocompromised patients, particularly those in intensive care or undergoing prolonged antibiotic treatment, also chronically colonizing cystic fibrosis patients (3, 6, 7). A recent meta-analysis reported a 40.5% mortality rate in *S. maltophilia* bacteremia cases (8).

The mechanisms underlying *S. maltophilia* pathogenesis remain relatively poorly understood. However, its ability to form biofilms on various surfaces significantly contributes to colonization, and the bacterium produces several extracellular enzymes that promote invasion, such as serine proteases, lipases, and cytotoxins (6, 7). Importantly, a major concern is *S. maltophilia*’s resistance to multiple antibiotics, which is largely intrinsic, involving low membrane permeability, efflux pumps, and antibiotic-modifying enzymes. Furthermore, the presence of inducible β-lactamases L1 and L2 in most isolates renders β-lactams, including carbapenems, largely ineffective (4, 9, 10). Despite its clinical relevance, *S. maltophilia* is ubiquitously found in the environment, including soil, water, sediments, sewage, as well as in association with animals (3, 11). In fact, the species (and other members of the *Stenotrophomonas* genus) is often found as the dominant member of microbial communities associated with plants, sometimes even engaging in beneficial interactions with them (12). Contrary to the closely related *Xanthomonas* and *Xylella* genera, *Stenotrophomonas* species are rarely reported as phytopathogens (12, 13). A large genomic study of about 1,300 *Smc* genomes from 22 countries identified 23 distinct clades (4). Four clades (Sgn1-4) contained mostly environmental lineages and were more phylogenetically distant from the remaining clades, while the other 19 included both clinical and environmental isolates. This suggests that *S. maltophilia* had an environmental origin, with multiple independent adaptations to pathogenicity. Furthermore, no specific genetic traits distinguished clinical from environmental strains, supporting the idea that its virulence traits evolved in the environment—a concept known as “coincidental evolution” (14).

*S. maltophilia* is frequently found in polymicrobial communities, both in clinical and environmental settings, co-existing with species like *Pseudomonas aeruginosa* and *Staphylococcus aureus* and many other bacterial genera such as *Burkholderia*, *Aeromonas*, *Bacillus*, and *Acinetobacter* (3, 7, 8, 15). Understanding its microbial interactions is essential for developing new therapeutic strategies, particularly as bacterial competition mechanisms play a crucial role in community dynamics (16–20). Among these mechanisms, secretion systems such as types IV, V, VI, and VII can mediate interbacterial competition (16, 21–26). The competitive arsenal of *S. maltophilia* remains largely unexplored, with functional characterization conducted in only a limited number of strains. An antibacterial type IV secretion system (T4SS), homologous to the system described in *X. citri* (25), has been implicated in bacterial competition in strain K279a (27–29). Furthermore, a recent study demonstrated a role for T6SS in antibacterial activity in one *S*. *maltophilia* cystic fibrosis isolate (30).

The type VI secretion system (T6SS) is a contractile molecular weapon widely distributed among Pseudomonadota (formerly known as Proteobacteria), where it plays a crucial role in interbacterial competition (31–34). Structurally, it consists of (i) a membrane complex anchoring the system to the bacterial envelope, (ii) a baseplate serving as a platform for assembly, and (iii) a contractile needle-like structure composed of an Hcp tube encased by a TssB/C sheath and tipped by VgrG and PAAR proteins. Upon sheath contraction, the Hcp tube and associated effectors are forcefully ejected, potentially puncturing a target cell, which can be prokaryotic or eukaryotic (31, 32, 35). T6SS effectors, which can be fused to structural components or recruited via adaptors, primarily target conserved cellular structures, such as the peptidoglycan layer, nucleic acids, membranes, and the cytoskeleton, leading to cell lysis or dysfunction (31, 35–38). To prevent self-intoxication, bacteria encode cognate immunity proteins, which are typically located next to their corresponding effector genes (39–42).

In this study, we investigated the prevalence, functionality, and effector diversity of T6SS clusters in *S. maltophilia* isolates from hospital and environmental sources in Brazil. Whole-genome sequencing was used to analyze the genomic diversity of these strains, as well as the characteristics and effector repertoire of their T6SS clusters. Functional assays demonstrated the role of T6SS in interbacterial competition and virulence in the *Galleria mellonella* larvae model.

## MATERIALS AND METHODS

### Bacterial strains and culture conditions

Twenty-nine clinical isolates and 13 environmental isolates of the *S. maltophilia* complex were analyzed. The clinical isolates were donated from the sample bank of the Microbiology Laboratory of Hospital de Clínicas at Unicamp by Prof. Carlos Emilio Levy (Faculdade de Ciências Médicas at Unicamp) (SisGen register number A78E9E5). The environmental isolates were provided by Prof. Eliana Guedes Stehling (Faculdade de Ciências Farmacêuticas de Ribeirão Preto/USP) and were first described in a study by Furlan et al. (43). Complete available information about all *S. maltophilia* strains is described in Table 1. *E. coli* DH5⍺ was used for cloning purposes; *E. coli* S17-λpir was used for conjugative transfer of the pEX18Tc plasmid to *S. maltophilia*; and the *E. coli* K-12 strain MG1655 *argA::*Tn10-Tet^R^ was used in interbacterial competition assays.

**TABLE 1:** Name, source of isolation, genogroup affiliation (when applicable), and identity confirmation of *Stenotrophomonas maltophilia* clinical and environmental isolates used in this study^*e*^^,^^*f*^

	Strain name	Source of isolation	Antimicrobial resistance profile					ID confirmed by PCR^*d*^	T6SS	Genogroup affiliation
			SXT	LFX	CAZ	MIN	Other			
1	S1	Blood	S	S	S	−	−	Yes	−	−
2	S2	Blood	S	S	R	−	−	Yes	−	−
3	S3	Blood	S	S	R	−	−	Yes	+	Sgn4
4	S4	Tip intravenous catheter	R	I	R	S	−	Yes	−	−
5	S5	Blood	S	S	S	−	−	Yes	−	−
6	S6	Blood	S	S	R	−	TOB - R	Yes	−	−
7	S7	Blood	S	S	I	−	−	Yes	−	−
8	S8	Ascitic fluid	S	S	S	−	−	Yes	-	-
9	S9	Blood	S	S	S	−	−	Yes	-	-
10	S10	Blood	S	S	R	−	−	Yes	+	Sgn4
11	S11	Oropharyngeal secretion	S	S	R	S	−	Yes	−	−
12	S12	Phlegm	S	S	R	S	−	Yes	−	−
13	S13	Oropharyngeal secretion	S	S	R	S	−	Yes	−	−
14	S14^*a*^	Sputum	R	R	R	S	−	Yes	−	−
15	S15	Sputum	R	S	S	S	−	Yes	−	−
16	S16	Oropharyngeal secretion	S	S	S	S	−	Yes	−	−
17	S17	Sputum	R	R	R	S	−	Yes	−	−
18	S18	Oropharyngeal secretion	S	S	R	S	−	Yes	−	−
19	S19	Sputum	S	S	S	S	−	Yes	−	−
20	S20	Oropharyngeal secretion	S	S	R	−	−	Yes	−	−
21	S21	Unknown	−	−	−	−	−	Yes	−	−
22	S22	Unknown	−	−	−	−	−	Yes	−	−
23	S23^*b*^	Unknown	R	R	R	S	TIM - S; CHL - R	Yes	−	−
24	S24	Unknown	−	−	−	−	−	Yes	−	−
25	S25	Blood	−	−	−	−	−	Yes	+	Sm15
26	S26	Blood	−	−	−	S	TIM - S	Yes	−	−
27	S27^*c*^	Blood	−	−	R	−	−	Yes	−	−
28	S28	Unknown	−	−	−	−	−	Yes	−	−
29	Sm17	Blood	−	−	−	−	−	Yes	−	−
30	S359	Banana (Teresina/PI)	R	R	R	S	−	Yes	−	−
31	S365	Cashew (Jardinópolis/SP)	R	S	R	S	−	Yes	−	−
32	S369	Landfill (Ribeirão Preto/SP)	R	R	R	S	−	Yes	−	−
33	S370	Landfill (Ribeirão Preto/SP)	R	R	R	S	−	Yes	−	Sm6
34	S371	Unknown (Jardinópolis/SP)	R	R	R	S	−	Yes	−	−
35	S377	Sunflower (Chapadão do Sul/MS)	R	R	R	S	−	Yes	+	ungrouped
36	S488	Coffee (Tabatinga/SP)	R	R	R	S	−	Yes	−	−
37	S489	Soy (Maringá/PR)	R	R	R	R	−	Yes	+	Sgn4
38	S490	Sugarcane (Tambaú/SP)	R	S	R	R	−	Yes	−	−
39	S491	Citrus (Água de Santa Bárbara/SP)	R	R	R	R	−	Yes	−	−
40	S493	Strawberry (Atibaia/SP)	S	S	R	R	−	Yes	−	−
41	S495	Soy (Jardinópolis/SP)	S	R	R	R	−	Yes	−	Sm10
42	S496	Sugarcane (Jardinópolis/SP)	S	R	R	R	−	Yes	−	−

^*a*^ Three colony sizes were perceived when this isolate was streaked onto TSA plates; each type of colony received a separate nomenclature (S14.1; S14.2; and S14.3).

^*b*^ Two colony sizes were perceived when this isolate was streaked onto TSA plates; each type of colony received a separate nomenclature (S23.1 and S23.2).

^*c*^ Two colony sizes were perceived when this isolate was streaked onto TSA plates; each type of colony received a separate nomenclature (S27.1 and S27.2).

^*d*^ Multiplex PCR and 23S rRNA PCR.

^*e*^ SXT: Sulfamethoxazole + Trimethoprim; LFX: Levofloxacin; CAZ: Ceftazidime; MIN: Minocycline; TOB: Tobramycin; TIM: Ticarcillin + Clavulanic Acid; CHL: Chloramphenicol.

^*f*^ +, present; −, absent or not known.

*S. maltophilia* was streaked on TSA plates (Tryptic Soy Agar; BD-Difco) before starting overnight cultures. Growth curves and interbacterial competition experiments were performed in TSB (Tryptic Soy Broth; Kasvi) and LB medium (10 g/L tryptone; 5 g/L yeast extract; 10 g/L NaCl; pH 7.5), respectively. Strains were routinely cultivated at 30°C (environmental strains) or 37°C (clinical strains and *E. coli*), with agitation (200 rpm). Tetracycline was used with DH5⍺ and S17-λpir for cloning purposes (12.5 μg/mL). Strains are described in Table 1 and Tables S1 and S3.

### Confirmation of *S. maltophilia* identity by *smeD/ ggpS* multiplex PCR

Overnight cultures in LB were grown from a single colony of each strain before stocking them with 40% glycerol at −80°C. Three strains (S14, S23, and S27) displayed different colony morphologies after being streaked on TSA plates. In these cases, individual overnight cultures were selected, and the strains were differentiated by name (for example, S14.1; S14.2, and S14.3).

A multiplex PCR strategy developed in the studies by Pinot et al. and Ribbeck-Busch et al. (44, 45) was used to confirm the identity of these clinical and environmental isolates as *S. maltophilia*. True *S. maltophilia* isolates should test positive for the 150-bp *smeD* fragment (SmeD is part of the SmeDEF efflux pump) and negative for the 885-bp *ggpS* fragment (glucosylglycerol phosphate synthase), which is diagnostic of *S. rhizophila*. Additionally, amplification of a 278-bp fragment from *23S rRNA* was used to support species identification and confirm DNA quality (46).

DNA was prepared directly from bacterial colonies, by suspending 200 µL of overnight cultures in 60 µL of ultrapure water, followed by boiling for 5 min. PCRs were performed in 25 µL reactions, using 0.06 U/ mL Easy Taq DNA polymerase (Transgen Biotech) in 1× reaction buffer (Mg_2_+ free) supplemented with 1.2 mM MgCl_2_ (LGC Biotecnologia), 25 μM of each dNTP, 1 μL of primers at 10 pmol (Table S2), and 2 μL of colony DNA. Colony DNA from strain *S. maltophilia* ATCC 1363 and *Xanthomonas citri*’s genomic DNA were used as positive and negative controls, respectively. PCR reactions were run as follows: 94°C initial denaturation, followed by 40 cycles of 94°C for 30 s, 58°C for 1 min, and 72°C for 1 min. Final extension was done at 72°C for 10 min.

### Identification of T6SS-positive strains by *tssC* degenerate PCR and Sanger sequencing

Identity-confirmed *S. maltophilia* isolates were tested for T6SS presence by amplification of the *tssC* gene using degenerate primers (Table S2). These primers were designed based on an alignment of *tssC* homologs found in the Xanthomonadales family and in *Stenotrophomonas* sp. genomes and enable the amplification of *tssC* variants from T6SS clades 3 and 4 (713-bp fragment) (47, 48). DNA quality was assessed by the amplification of *23S rRNA* (Table S2). A positive control for *tssC* amplification (*X. citri*’s genomic DNA) and *23S* amplification (*S. maltophilia* ATCC13637’s genomic DNA) was included in the reactions. PCR reactions were run as described in the previous section, with adaptations in melting temperatures and extension time according to the primers and amplicon size, respectively. DNA bands corresponding to the expected size of the *tssC* amplicon (from *tssC* positive samples) were excised from agarose gels and purified using GeneJET Gel Extraction Kit (Thermo Fisher Scientific). Purified PCR products were Sanger sequenced (EXXTEND, Brazil), and *tssC* identity was confirmed by BLAST.

### Whole-genome sequencing

*S. maltophilia* isolates confirmed to have a *tssC* homolog were selected for whole genome sequencing. Genomic DNA was extracted from saturated liquid cultures using the Wizard Genomic DNA purification kit (Promega), following the manufacturer’s instructions. DNA quantification was performed using Qubit, and DNA integrity was analyzed by agarose gel electrophoresis. Samples were sent to MicrobesNG (Birmingham, UK) or CEFAP (University of São Paulo, Brazil) for WGS sequencing. At MicrobesNG, libraries were sequenced on an Illumina NovaSeq 600 using a 250 bp paired-end protocol. Reads were trimmed using Trimmomatic version 0.30 (49). *De novo* genome assembly was performed using SPAdes version 3.7 (50). At CEFAP, genomes were sequenced on an Illumina NextSeq 500 using a 75 bp paired-end protocol, and assembly was performed using Unicycler v0.5.1 (51). All genomes were annotated using the Prokaryotic Genome Annotation Pipeline (PGAP) (52) when the contigs were deposited to the NCBI (see Data Availability section).

### Phylogenetic analysis

#### Genome selection

A total of 1,520 *Stenotrophomonas* genomes available in GenBank as of May 2024 were downloaded and filtered to exclude highly fragmented assemblies, retaining only those with a maximum of 100 contigs to minimize the risk of low completeness or contamination. These genomes were compared with the isolates S25, S3, S10, S377, S370, S495, and S489 using fastANI (53). For each isolate, up to five genomes were selected, considering only those with an average nucleotide identity (ANI) value of at least 95%, the established threshold for species-level identity (53). Additionally, genomes from *S. maltophilia* NCTC 10257 (reference and complete RefSeq genome from type strain *S. maltophilia* ATCC 13637), the antibacterial T4SS-containing *S. maltophilia* K279a, and representatives of the T6SS i1 and i4 subgroups (*Stenotrophomonas* sp. KCTC and *Stenotrophomonas* sp. LM091, respectively, were included. This stage yielded 28 public *Stenotrophomonas* genomes (described in Tables S5 and S6), resulting in a data set with 35 genomes.

#### Phylogenetic tree

The 35 selected *Stenotrophomonas* genomes, along with *X. citri* pv. *citri* A306 (used as an outgroup due to its well-characterized T6SS and T4SS systems), were analyzed for homologous gene clustering using Get_Homologues (54). The OrthoMCL algorithm (-M option) was applied with identity and query coverage thresholds of 80%. Following the initial clustering step, the *compare_clusters.py* script was used to obtain the homologous and unique genes shared across all genomes. Each gene family was subsequently aligned using MAFFT (55) with a maximum of 1,000 iterations. Alignment regions with excessive gaps were manually curated and removed. Finally, a Maximum Likelihood phylogenetic tree was inferred using IQtree2 (56), with 1,000 bootstrap replicates and a substitution model selected by ModelFinder (57).

### *S. maltophilia* complex analyses

#### Identification of the isolates

The 16S rRNA sequences were extracted from the isolates as well as from *S. maltophilia* NCTC 10257 and compared using BLASTn. Genomes were assigned to the *S. maltophilia* complex if their 16S rRNA sequences shared at least 99% identity with a type strain from *S. maltophilia* species, following the methodology of (4).

#### Comparisons with the 23 monophyletic lineages

The *Stenotrophomonas* genomes included in the phylogenetic tree were compared to the 23 monophyletic lineages (Sgn1–Sgn4, Sm1–Sm18) described by Gröschel et al. (4). Of the 1,520 *Stenotrophomonas* genomes analyzed, all previously defined lineages were represented except for Sm13, Sm15, Sm16, and Sm17. These lineages lacked publicly available assembled genomes and required *de novo* assembly from raw sequencing data. The corresponding sequencing data (ERR3300126, ERR3299978, ERR3309056, and ERR3300026, representing Sm13, Sm16, Sm15, and Sm17, respectively) were retrieved using *fasterq-dump* and assembled with SPAdes v4.0.0 (50). The assembled genomes were subsequently compared to phylogenetic tree genomes using fastANI. A genome was assigned to a specific monophyletic lineage if it exhibited at least 95% ANI with a representative genome of that lineage.

### Identification of T6SS and T4SS clusters

The presence of T6SS and antibacterial T4SS clusters was investigated in the isolates S25, S3, S10, S377, S370, S495, and S489, as well as in the 28 *Stenotrophomonas* genomes included in the phylogenetic analysis. The searches were performed using a set of protein sequences corresponding to TssB, TssC, TssE, TssF, and TssG (for T6SS clusters) and VirD4 and VirB4 (for antibacterial T4SS clusters). Given the diversity of described T6SS clusters, three distinct T6SS types were analyzed using representative TssBCEFG protein sequences from the i1, i3, and i4 clades. The identification of these clusters in the genomes was performed using *tBLASTn* alignments, applying a minimum threshold of 80% identity and query coverage for each protein sequence within the cluster.

### Identification of conserved domains in predicted T6SS effectors

Putative effector/immunity pairs associated with VgrG proteins in the main T6SS cluster (i4 clade) from all *S. maltophilia* T6SS+ isolates were investigated. To identify the domains involved in their potentially toxic activity, we combined information given by gene annotations with protein sequence searches using the NCBI’s Conserved Domain Database (CDD) (NCBI Conserved Domain Search, https://www.ncbi.nlm.nih.gov/Structure/cdd/wrpsb.cgi) (58).

### Recombinant DNA techniques

Genetic manipulation and molecular cloning followed previously established methods. PCR amplifications were performed using FastPol HF or TaqPol polymerases (CellCo), unless otherwise stated, following the manufacturer’s recommendations. PCR products were purified using GeneJET PCR Purification kit (Thermo Fisher Scientific), according to the supplier’s protocol. Plasmids were purified by alkaline lysis method (miniprep) *in house* or using GeneJET Plasmid Miniprep kit (Thermo Fisher Scientific), following the manufacturer’s recommendations. FastDigest restriction enzymes and T4 ligase (Thermo Fisher Scientific) were used according to the supplier’s protocols.

In-frame deletions of *tssM* in *S. maltophilia* isolates were obtained by a two-step integration/excision exchange using the pEX18Tc vector (59) (Table S4). Fragments of 1-kb flanking the *tssM* gene of strain *S. maltophilia* SM3 were amplified by PCR and cloned into the XbaI and HindIII sites of the pEX18Tc vector. The pEX18Tc-Δ*tssM* vector was checked by Sanger sequencing (LACTAD, Unicamp). Sequences were verified using SnapGene version 1.1.3 and Geneious Prime version 2024.0.3.

The plasmid was transformed into *E. coli* S17-λpir by electroporation (60) and then transferred to *S. maltophilia* strains by conjugation, following the protocol described by Milton et al. (61) with slight modifications. Briefly, *S. maltophilia* and *E. coli* S17-λpir were grown to mid-logarithmic phase. Cells were harvested by centrifugation, washed and concentrated two times using LB medium. Recipient and donor strains were mixed at a 1:5 ratio and spotted on LB agar plates at 37°C for 24 h. Mating spots were scooped and restreaked (or diluted in LB medium and plated) on LB plates containing tetracycline (20 μg/mL) and gentamicin (10 μg/mL) to select *S. maltophilia* strains carrying the integrated plasmid. Positive colonies were restreaked on a fresh plate with antibiotics to confirm selective growth and then grown overnight in LB without antibiotics to enable intramolecular recombination. For *sacB-*based counterselection, cultures were restreaked on LB plates supplemented with 10% sucrose (62) without antibiotics and incubated overnight at 37°C. Sucrose-resistant colonies were restreaked in parallel on LB with 10% sucrose and LB with tetracycline (20 μg/mL) to confirm loss of the pEX18Tc plasmid (suc^R^/Tet^S^). Mutant clones were confirmed by colony PCR, using the primers listed in Table S2.

### Interbacterial competition assays

Co-culture assays between *S. maltophilia* and *E. coli* (MG1655- *argA::*Tn10-Tet^R^) were performed following the methodology described in (30), with minor modifications. Briefly, overnight cultures were grown at 37°C or 30°C and 200 rpm, then diluted 1:100 in fresh LB medium. Cultures were grown for 4 h, after which the cells were collected by centrifugation and adjusted in LB to an OD_600_ of 1 for *S. maltophilia* and 0.5 for *E. coli*. Each *S. maltophilia* strain (volume 1×) was mixed with *E. coli* (volume 10×) (thus generating a 20:1 ratio). The mixture was centrifuged, and the pellet was resuspended in 1× volume of LB. 5 µL of each mixture was applied onto a piece of filter paper placed on an LB plate. Plates were incubated at 37°C or 30°C for 20 h, when the filter papers were resuspended in 1 mL LB. Co-cultures were serially diluted and 5 µL of each dilution was spotted onto LB and LB with tetracycline (20 µg/mL) plates for enumeration of CFU/mL.

### Growth curves

Saturated cultures were diluted to an OD_600_ of 0.05 (clinical) or 0.1 (environmental) in TSB medium and grown in the shaker at 37°C or 30°C, with agitation (200 rpm). Absorbance (OD_600_) of cultures was measured every hour for 5–6 h.

### Virulence assays with *Galleria mellonella*

Virulence assays using *G. mellonella* were performed as previously described (63–65). Saturated overnight cultures were diluted and grown to exponential phase in LB medium. An inoculum of 10⁷ CFU was injected into each *G. mellonella* larva (larvae weighed between 200 and 350 mg). Each experimental group consisted of 10 larvae, while a control group of five larvae was inoculated with PBS solution. Clinical isolates (S3, S10, and S25) and their respective Δ*tssM* mutants were prepared for inoculation and larvae were incubated at 37°C. In the case of environmental isolates (S377 and S489), cultures were grown, and larvae were incubated both at 28°C and 37°C. For all tested strains, inoculated larvae were checked every 24 h, when their mortality was scored, for a total of 96 h. K279a strain was used as a control in all experiments. Experiments were repeated at least three times. Survival curves were compared in GraphPrism, and complete statistical analyses are provided in Table S7.

## RESULTS

### Detection of T6SS-positive *S. maltophilia* strains and identification of T6SS and T4SS clusters

Twenty-nine clinical strains initially identified as *S. maltophilia* using various culture-based methods at Hospital de Clínicas from Unicamp (Campinas, Brazil) along with 13 environmental isolates previously identified as *S. maltophilia* based on MALDI-TOF VITEK MS (bioMérieux, Inc., Durham, NC) and *23S rRNA* sequencing (43) were further confirmed using a multiplex PCR-based strategy (44, 45). True *S. maltophilia* isolates tested positive for the amplification of a 150-bp fragment of the *smeD* gene (from SmeDEF efflux pump) and negative for the amplification of a 885-bp fragment of the *ggpS* gene (glucosylglycerol phosphate synthase), which is diagnostic of *S. rhizophila*. An additional confirmation step was performed by the amplification of a 278-bp fragment from the *23S rRNA* gene (46). Complete available information on all *S. maltophilia* isolates is given in Table 1.

Identity-confirmed *S. maltophilia* isolates were tested for T6SS presence by amplification of the *tssC* gene using degenerate primers designed to amplify *tssC* from T6SS clades i3 and i4 (47, 48). Re-amplification of the 278-bp *23S rRNA* fragment was performed in parallel to attest colony DNA quality.

Degenerate *tssC* PCR results indicated that three clinical (S3, S10, and S25; prevalence of 10.3%) and two environmental *S. maltophilia* isolates (S377 and S489; prevalence of 15.4%) contained a *tssC* gene and, possibly, a T6SS cluster (Fig. S1 and S2). The complete genome sequences of the five *tssC*-positive isolates (S3, S10, S25, S377, and S489) and two *tssC*-negative strains, S495 and S370, were determined.

**Fig 1:**
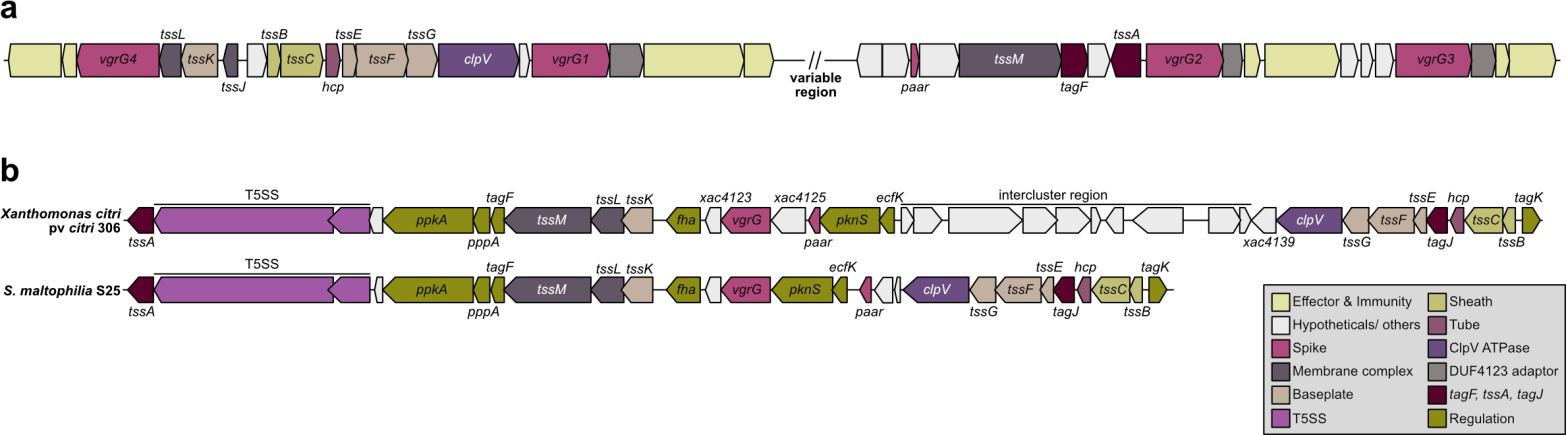
T6SS clusters found in *S. maltophilia* isolates. (**a**) Schematic representation of the general backbone of i4-clade T6SS cluster found in all *S. maltophilia* isolates (clinical S3, S10, and S25; environmental S377 and S489). Effector/immunity pairs of each strain are discussed in Fig. 5. (**b**) Schematic representation of the additional i3-clade T6SS cluster from strain S25. This cluster is akin to the anti-eukaryotic T6SS found in *X. citri* pv. *citri* 306. Both clusters are depicted for comparison. In all cases, genes are color-coded according to the components of the T6SS machinery associated with their predicted proteins (described in the legend in the gray box).

T6SS clusters have been previously classified into four main subtypes (T6SS^i-iv^). T6SS^i^, found in the Pseudomonadota phylum, corresponds to the majority of known clusters and is subdivided into five phylogenetic clades (i1-i5) (66). *S. maltophilia* belongs to the Xanthomonadales order, which includes human and plant pathogens as well as environmental bacteria. An *in silico* analysis of 71 Xanthomonadales genomes from the KEGG database identified 35 genomes with one or more T6SS clusters, belonging to clades i1, i3, and i4 (47). Following our initial PCR-based screenings, we searched for the T6SS clusters in the five sequenced *S. maltophilia* genomes. Sequence searches were performed using tBLASTn, employing a set of protein sequences from the *tssBCEFG* genes from T6SS subtypes i1, i3, and i4. This search identified a conserved T6SS cluster backbone in all strains, akin to the cluster described in (30), from the i4 clade (Fig. 1a). Notably, an additional T6SS cluster was identified in strain S25, which resembled the anti-eukaryotic i3-clade T6SS cluster found in *X. citri* (47, 67)(Fig. 1b).

**Fig 2:**
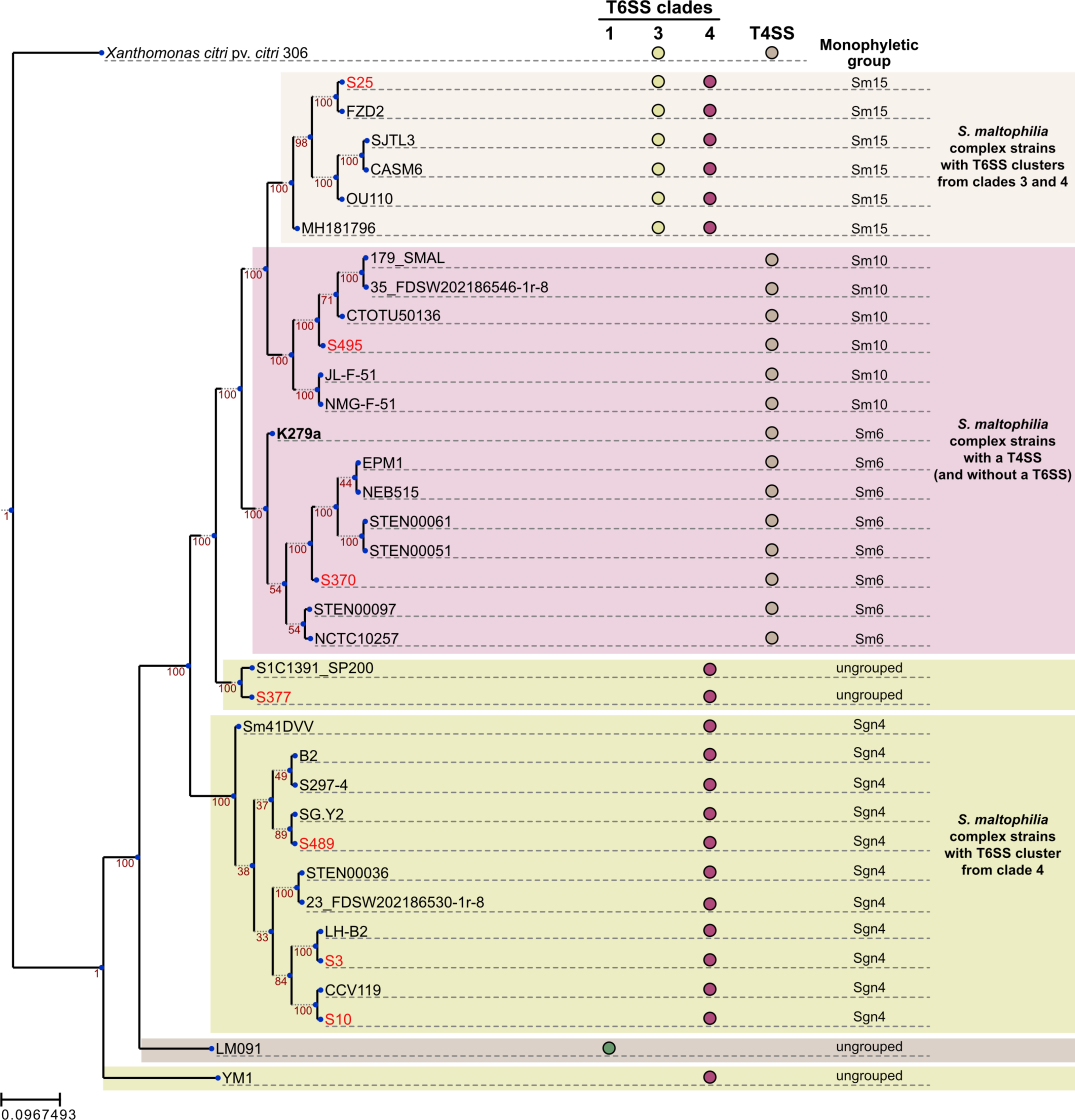
Phylogenetic diversity and distribution of T6SS and T4SS clusters in *S. maltophilia* isolates. A maximum-likelihood phylogenetic tree was reconstructed from a set of 36 genomes, including seven from this study (highlighted in red), 24 GenBank genomes that share at least 95% ANI with one or more of the seven initial genomes, and genomes from type strain *S. maltophilia* NCTC 10257, *S. maltophilia* K279a, *Stenotrophomonas* sp. KCTC and *Stenotrophomonas* sp. LM091. *X. citri* pv. *citri* A306 was used as the outgroup. Three major clades were formed based on the presence of T6SS and T4SS clusters (color-coded), which are indicated by small colored circles and legends on the right. Monophyletic groupings according to (4) are also displayed.

*S. maltophilia* K279a, a blood isolate that has been sequenced and more thoroughly investigated (68), does not have a T6SS cluster, harboring an antibacterial T4SS instead. This type of secretion system was originally identified and characterized in *X. citri* (25), and bacterial killing activity in *S. maltophilia* K279a has been demonstrated against *E. coli, X. citri*, and *P. aeruginosa* (27–29). To search for the presence of the antibacterial T4SS, we surveyed our sequenced *S. maltophilia* genomes using tBLASTn to identify homologs of the *X. citri* T4SS proteins VirB4 and VirD4, which correspond, respectively, to a highly conserved T4SS component and an essential T4SS protein that display higher variability among different T4SS subgroups (25). Interestingly, only strains S370 and S495, which were sequenced but did not actually contain T6SS clusters, harbored a T4SS (Fig. 2).

### Phylogenetic analysis

Our final set of *S. maltophilia* strains with genomes fully sequenced contained three clinical strains (S3, S10, and S25) and four environmental isolates (S370, S377, S489, and S495). As previously described, these strains contain T6SS clusters or, in the case of S370 and S495, an antibacterial T4SS cluster. Considering the high genomic variability that is characteristic of the *S. maltophilia* complex (4), we investigated the level of phylogenetic relatedness of our strains and their position within a *S. maltophilia* phylogenetic tree.

To explore the genetic diversity of these isolates, we conducted an ANI analysis using all publicly available *Stenotrophomonas* genomes from GenBank, retaining the most similar genomes to each new isolate (Tables S5 and S6). Additionally, we evaluated the genomic similarity of our isolates with strains of particular interest, including the *Stenotrophomonas* type strain NCTC 10257, the antibacterial T4SS-harboring *S. maltophilia* K279a, and species containing i1 and i4 T6SS clusters (*Stenotrophomonas* sp. KCTC and *Stenotrophomonas* sp. LM091, respectively).

Notably, isolate S377 exhibited low relatedness to other *Stenotrophomonas* genomes, displaying more than 95% ANI with only one genome, which was labeled in the GenBank record as *uncultured Stenotrophomonas* (accession number GCF_913778665.1) (Table S6). Further analyses also revealed that this isolate is the only one in our final set that cannot be assigned to any of the 23 monophyletic lineages established by Gröschel et al. (4) (Fig. 2). This finding suggests that S377 may represent a novel lineage within the *S. maltophilia* complex.

Thirty-six genomes (corresponding to our seven isolates, 28 public genomes, and the *X. citri* A306 outgroup genome) were used to infer a phylogenetic tree. A total of 684 homologous genes were identified using the *Get_Homologues* pipeline (https://github.com/eead-csic-compbio/get_homologues), followed by the construction of a Maximum-Likelihood phylogenetic tree (Fig. 2). The analysis revealed the separation of the isolates into five major clades, showing that S3, S10, and S489 were placed in the same clade, whereas S25, S377, S370, and S495 were placed each in a different clade.

Next, the T6SS and antibacterial T4SS were identified in the genomes used for phylogenetic reconstruction. Three T6SS subtypes were investigated: i1, i3, and i4. For both systems, sequence searches were performed using tBLASTn, employing a set of protein sequences from the *tssBCEFG* genes as queries for T6SS and the *X. citri virB4* and *virD4* genes as queries for antibacterial T4SS. The results, which are also displayed in the phylogenetic tree (Fig. 2), reveal a clear separation of secretion systems across three major clades: one consisting of genomes harboring only the i4 T6SS (S377, S3, S10, and S489); a second comprising genomes carrying both i4 and i3 T6SS (S25); and a third consisting of genomes containing only the T4SS (S495 and S370).

Finally, we added to the phylogenetic tree information about the 23 monophyletic lineages established by Gröschel et al. (4). We observed consistency between the lineages and the clades in our tree, in agreement with the results by Gröschel et al. (4).

### *S. maltophilia* isolates display variable levels of interbacterial competition

**Fig 3:**
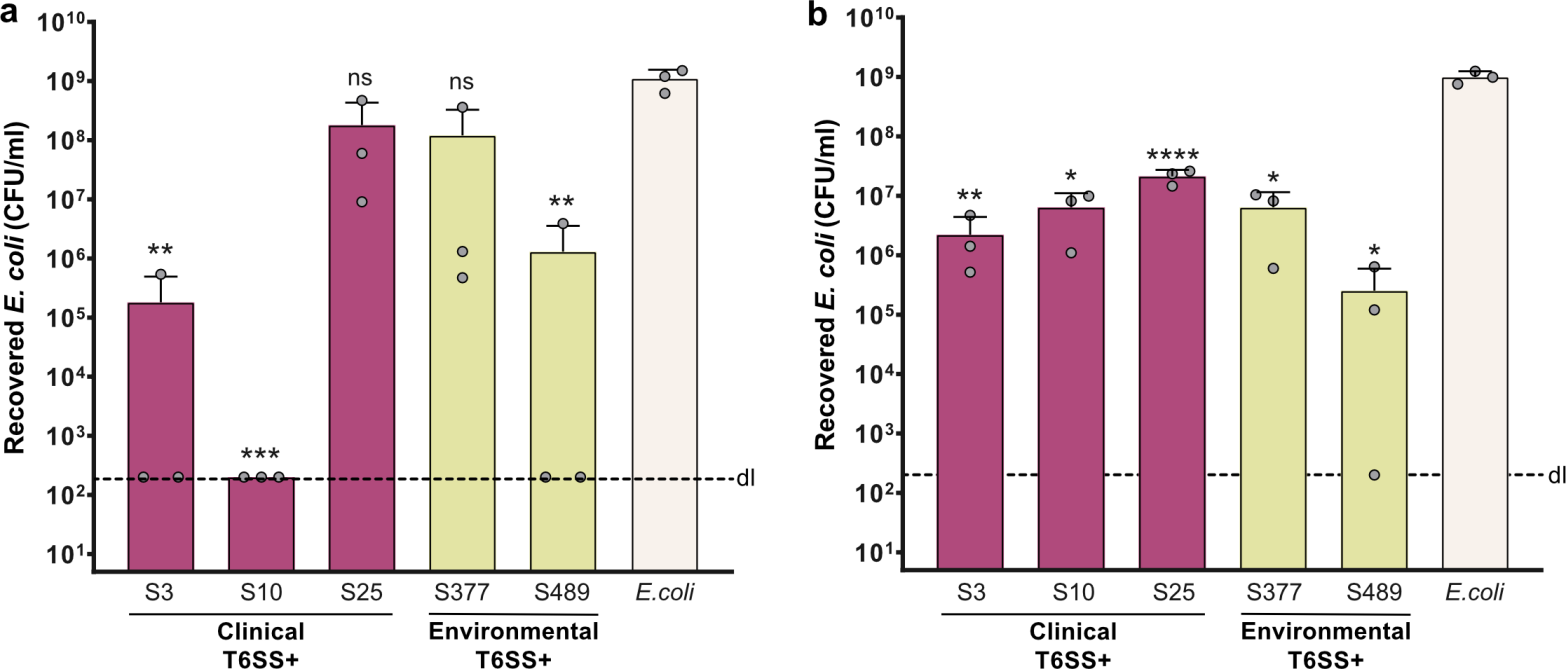
*S. maltophilia* isolates present various levels of interbacterial competition ability. Bacterial killing assays using *E. coli* as prey. *S. maltophilia* strains and *E. coli* prey were mixed at a 20:1 ratio, spotted onto LB agar plates, and incubated for 20 h at 37°C (**a**) or 30°C (**b**). The number of surviving prey is depicted on the Y-axis (CFU/mL). Bar plots in both panels represent the average of three independent biological replicates (±SD), which are indicated by small gray circles. dl, detection limit. *S. maltophilia* isolates are colored according to their sampling origin (clinical strains in pink, environmental strains in light green), while the control of *E. coli* alone is shown in beige. Statistical significance is shown using one-way ANOVA (**a**) or Welch ANOVA (**b**), followed by Dunnett’s multiple comparisons test. In both graphs, strains were compared to *E. coli*, and significance is displayed above each column. *, *P* < 0.05; **, *P* < 0.01; ***, *P* < 0.001; ****, *P* < 0.0001; ns, not significant.

We then focused on our set of T6SS-positive strains, aiming to evaluate the interbacterial killing potential of these isolates. As shown in Fig. 3a, these strains were able to eradicate *E. coli* prey at various levels of efficiency. Remarkably, clinical isolates S3 and S10 and the environmental strain S489 killed *E. coli* quite potently. On the other hand, the clinical isolate S25 and the environmental S377 strain displayed a negligible killing activity in comparison with the other strains (Fig. 3a).

Next, we tested whether the different levels of killing potential of *S. maltophilia* isolates could be explained by a regulatory mechanism responsive to different growth conditions. Since *S. maltophilia* is considered an environmental bacterium and an occasional human pathogen, we decided to test the effects of growth temperature on bacterial competitiveness. For that, we performed the competition experiments with *E. coli* prey at 30°C (instead of 37°C). As can be seen in Fig. 3b, the clinical isolates S3 and S10 killed *E. coli* less efficiently at 30°C than at 37°C. Furthermore, with the environmental isolate S489, two out of three replicates at 37°C rendered prey below detection level (Fig. 3a). At 30°C, on the other hand, one replicate fell below detectable levels, while the other two had prey recovered to around 10^5^–10^6^ CFU/mL (Fig. 3b). This slight change in phenotype might simply suggest experimental variation. Importantly, strains S3 and S10 grow less efficiently when cultured at 30°C compared to 37°C (Fig. S3a and b). Strain S489, however, grows approximately at the same rate both at 30°C and 37°C (Fig. S3f). Nevertheless, recovery of both prey and predator bacteria from competition experiments was similar in both temperatures (Fig. S4b), suggesting that poor growth is not the explanation for the differences observed.

In the case of clinical strain S25 and environmental isolate S377, competition experiments at 30°C displayed increased killing activity (Fig. 3b). Approximately 10-fold to 100-fold less *E. coli* prey was recovered after incubation with S25 and S377 at 30°C compared to the prey alone (Fig. 3; Fig. S4a). Considering that these isolates grow very similarly in both temperatures (Fig. S3c and e), these results suggest that incubation at lower temperatures might favor their T6SS activity, either through better activation of the whole system or better functioning of certain effectors.

### The T6SS is required for killing activity in clinical isolates

**Fig 4:**
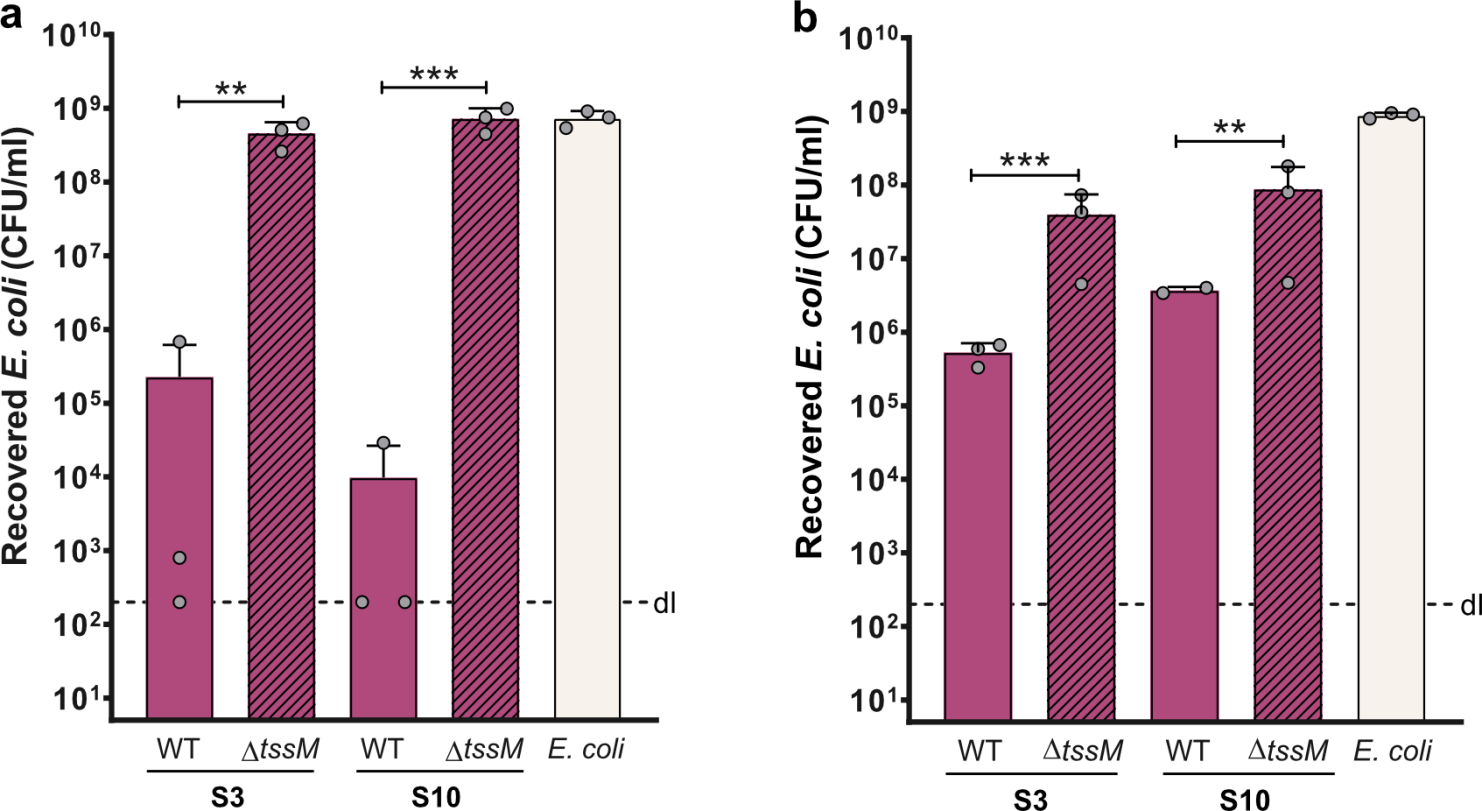
T6SS requirement for interbacterial competition ability in clinical *S. maltophilia* isolates. Bacterial killing assays using *E. coli* as prey. *S. maltophilia* strains and *E. coli* prey were mixed at a 20:1 ratio, spotted onto LB agar plates, and incubated for 20 h at 37°C (**a**) or 30°C (**b**). The number of surviving prey is depicted on the Y-axis (CFU/mL). Bar plots in both panels represent the average of three or two independent biological replicates (±SD), which are indicated by small gray circles. dl, detection limit. Clinical *S. maltophilia* isolates are colored in pink, with *tssM* mutant strains marked by dashed lines, while the control of *E. coli* alone is shown in beige. Statistical significance was assessed by comparing each strain to its ∆*tssM* counterpart, using one-way ANOVA followed by Tukey’s (**a**) or Sidak’s (**b**) multiple comparisons tests. **, *P* < 0.01; ***, *P* < 0.001.

To determine whether the observed interbacterial killing was dependent on the T6SS, we deleted the *tssM* gene—which encodes an essential component of the T6SS membrane complex—in strains S3 and S10, both of which exhibited the highest antibacterial activities in our *E. coli* competition assays. Notably, *E. coli*’s effacement was completely abrogated in the Δ*tssM* mutants, indicating that the observed interbacterial killing was dependent on T6SS activity (Fig. 4a). The results from competition assays performed at 30°C also showed that the residual killing ability under this growth condition was significantly diminished in the *tssM* mutant strains (Fig. 4b). However, unlike what was observed at 37°C, the number of prey cells recovered was still lower than in the *E. coli*-only control. Interestingly, Crisan et al. (30) observed the same trend in their experiments at 37°C using one *S*. *maltophilia* isolate. That might be explained by an additional competition mechanism that could be activated at 30°C in these strains. These results further suggest that the T6SS of isolates S3 and S10 have reduced activity at lower temperatures, which will be investigated in future studies.

### *S. maltophilia* isolates and their T6SS-associated effector diversity

The observed variability in interbacterial killing potential of our set of *S. maltophilia* isolates could be explained by the T6SS effector repertoire of these strains. T6SS effectors are usually associated with the needle components VgrG, Hcp, or PAAR, where they can be found as fused domains. Alternatively, effectors can be recruited to these components as cargo through adaptors, which can either be domains of the needle components themselves or independent proteins (31, 35). Considering that the T6SS i4 cluster common to all evaluated *S. maltophilia* isolates contains four encoded *vgrG* genes, we investigated the predicted domains present in the proteins encoded by genes located downstream of these *vgrG* genes. Importantly, it has been recently shown that *S. maltophilia* and also *S. rhizophila* can have multiple extra *vgrG* genes scattered throughout their genomes (30, 69). Although we could observe the presence of extra *vgrG* copies in our set of strains, we did not seek to characterize these genes and the possibly associated effectors in the present study.

**Fig 5:**
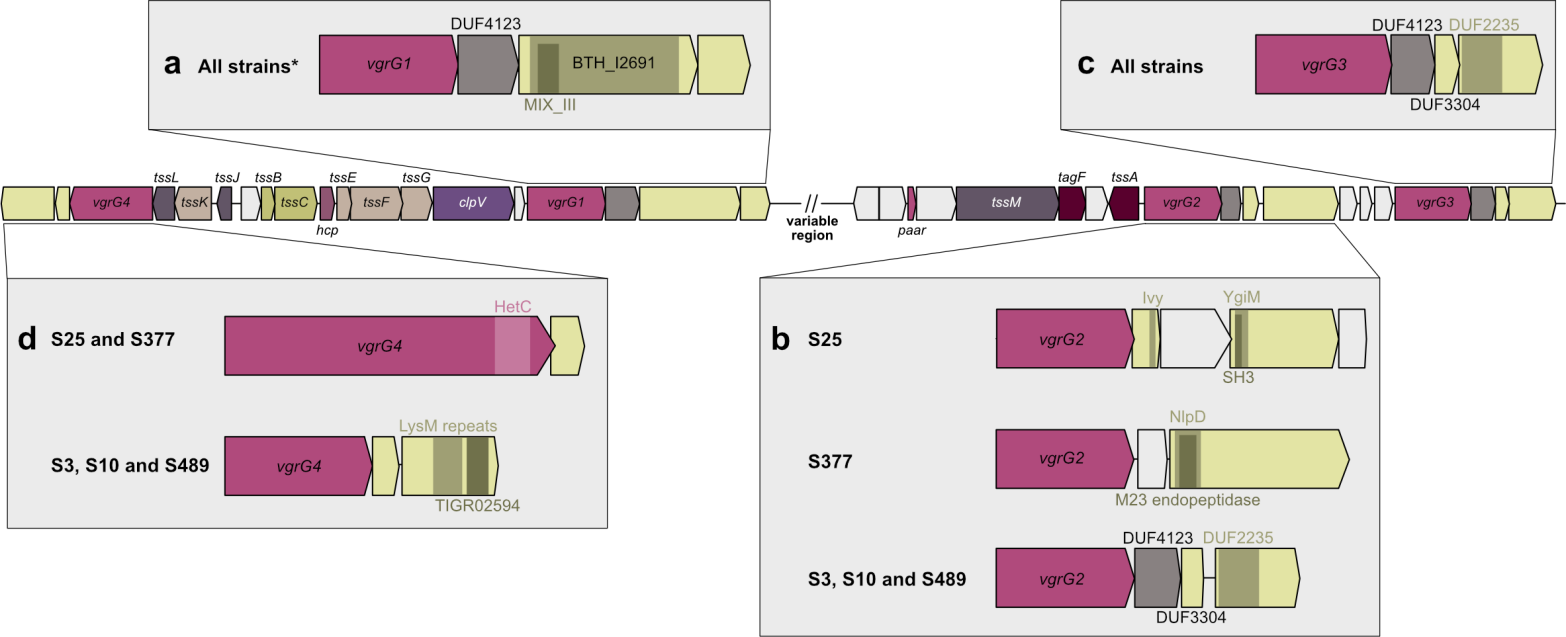
Diversity of effectors associated with a conserved T6SS cluster in *S. maltophilia* strains. A conserved T6SS i4 cluster backbone is found in clinical and environmental *S. maltophilia* isolates. The cluster encodes four VgrGs, each associated with a diverse set of effector/immunity pairs. *vgrG* genes are numbered according to Reference 30, and are shown in panels **a–d**. Domains found in predicted proteins are indicated by colored rectangles and legends. Genes encoding proteins with unknown function are colored in light gray. * It was not possible to undoubtedly identify *vgrG1* and its associated effector/immunity pair in strain S489.

In the case of *vgrG1*, all strains (except for S489, for which we could not undoubtedly identify this specific *vgrG*) had *vgrG* followed by a gene encoding a DUF4123 domain, typically found in adaptors (36, 70), then a gene with a BTH_I2691 domain and a smaller gene without predicted domains (Table 2; Fig. 5a). Importantly, a search for the BTH_I2691 domain in the genome of strain S489 did not find any matches, suggesting that this strain contains a different effector in this position or that this portion of this strain’s genome was lost during assembly. T6SS effectors containing a BTH_I2691 domain are known to work as pore-forming toxins, which create channels in prokaryotic or eukaryotic target cellular membranes, disrupting their chemiosmotic gradients (38, 71). The smaller downstream gene probably encodes the cognate immunity protein.

**TABLE 2:** Predicted domains found in putative effectors associated with VgrGs encoded in the main T6SS i4-cluster in *S. maltophilia* isolates

	S3	S10	S25	S377	S489
VgrG1	BTH_I2691 (pore forming)	BTH_I2691 (pore forming)	BTH_I2691 (pore forming)	BTH_I2691 (pore forming)	Undefined
VgrG2	DUF2235 (phospholipase)	DUF2235 (phospholipase)	YgiM/ SH3 (peptidoglycan degradation?)	NlpD/ M23 endopeptidase	DUF2235 (phospholipase)
VgrG3	DUF2235 (phospholipase)	DUF2235 (phospholipase)	DUF2235 (phospholipase)	DUF2235 (phospholipase)	DUF2235 (phospholipase)
VgrG4	TIGR02594 (NlpC/P60 super family) (peptidoglycan degradation)	TIGR02594 (NlpC/P60 super family) (peptidoglycan degradation)	Evolved VgrG with a Het-C domain (unknown function)	Evolved VgrG with a Het-C domain (unknown function)	TIGR02594 (NlpC/P60 super family) (peptidoglycan degradation)

VgrG2-associated effectors were highly diverse among the isolates. Strains S3, S10, and S489 were all equipped with a DUF4123-containing adaptor, followed by a DUF3304-containing immunity protein and a DUF2235 effector (Table 2; Fig. 5b). DUF2235 is an uncharacterized alpha/beta hydrolase domain, previously associated with T6SS phospholipases in *P. aeruginosa* and *E. coli*, for example (72, 73). In the case of the clinical isolate S25, *vgrG2* was followed by a set of four genes in a putative operon. The first encoded protein contains a predicted Ivy domain (for “Inhibitor of vertebrate lysozyme”). The second and fourth encoded proteins have no predicted domains, while the longer, third protein possesses YgiM and SH3 domains in the N-terminus (Table 2; Fig. 5b). SH3 domains are peptide-binding modules widely encountered in bacteria and commonly found fused to several NlpC/P60 domains of cell wall hydrolases and other cell wall modules, presumably being involved in peptidoglycan binding (74). This domain has also been found in TagV, a lipoprotein from the T6SS of *Serratia marcescens* that is proposed to tether the baseplate to the cell wall or sense cell wall status (75). Finally, VgrG2 in the environmental isolate S377 is associated with a 1,200 amino acid-long protein containing a predicted NlpD domain in the N-terminus and is annotated as an M23 endopeptidase. These proteins are metallopeptidases that cleave peptidoglycan, indicating their activity targeting the bacterial cell wall. The upstream gene probably encodes the corresponding immunity, which does not present any conserved domain.

VgrG3-associated proteins were the most well-conserved in our strains (Table 2; Fig. 5c), not only considering the predicted domains but also at the sequence level. All strains have VgrG3 equipped with a DUF4123 adaptor, a DUF3304 immunity, and a DUF2235 phospholipase effector. This same adaptor/immunity/effector trio was found at this *vgrG* in *S. maltophilia* STEN00241 (30) and also in *S. rhizophila* CFBP13503 (69). This particular effector-equipped VgrG might therefore be a “core T6SS effector” of *Stenotrophomonas* carrying this type of cluster, as was proposed for *P. aeruginosa*’s effector repertoire (76). This same set of proteins is also found associated with VgrG2 in strains S3, S10, and S489, as previously described (Fig. 5b).

Finally, in the case of VgrG4, strains S3, S10, and S489 again harbored the same associated effector/immunity pair. *vgrG4* is followed by a shorter gene that most likely encodes the immunity protein. The following predicted effector contains LysM repeats in the central part of the protein and a TIGR02594 domain (NlpC/P60 superfamily) in its C-terminus. NlpC/P60 is a diverse family of peptidoglycan peptidases (77). Members of this family have been associated with the T6SS of *P. aeruginosa* and *V. cholerae*, for example, and are involved in cell wall degradation (78, 79). Finally, strains S25 and S377 both presented an evolved VgrG4, which contained a C-terminal uncharacterized HetC domain. This domain is also found in bacterial polymorphic toxins and is proposed to have phospholipase or metal-dependent nuclease activity (80).

### T6SS-mediated virulence of *S. maltophilia* isolates against *G. mellonella*

**Fig 6:**
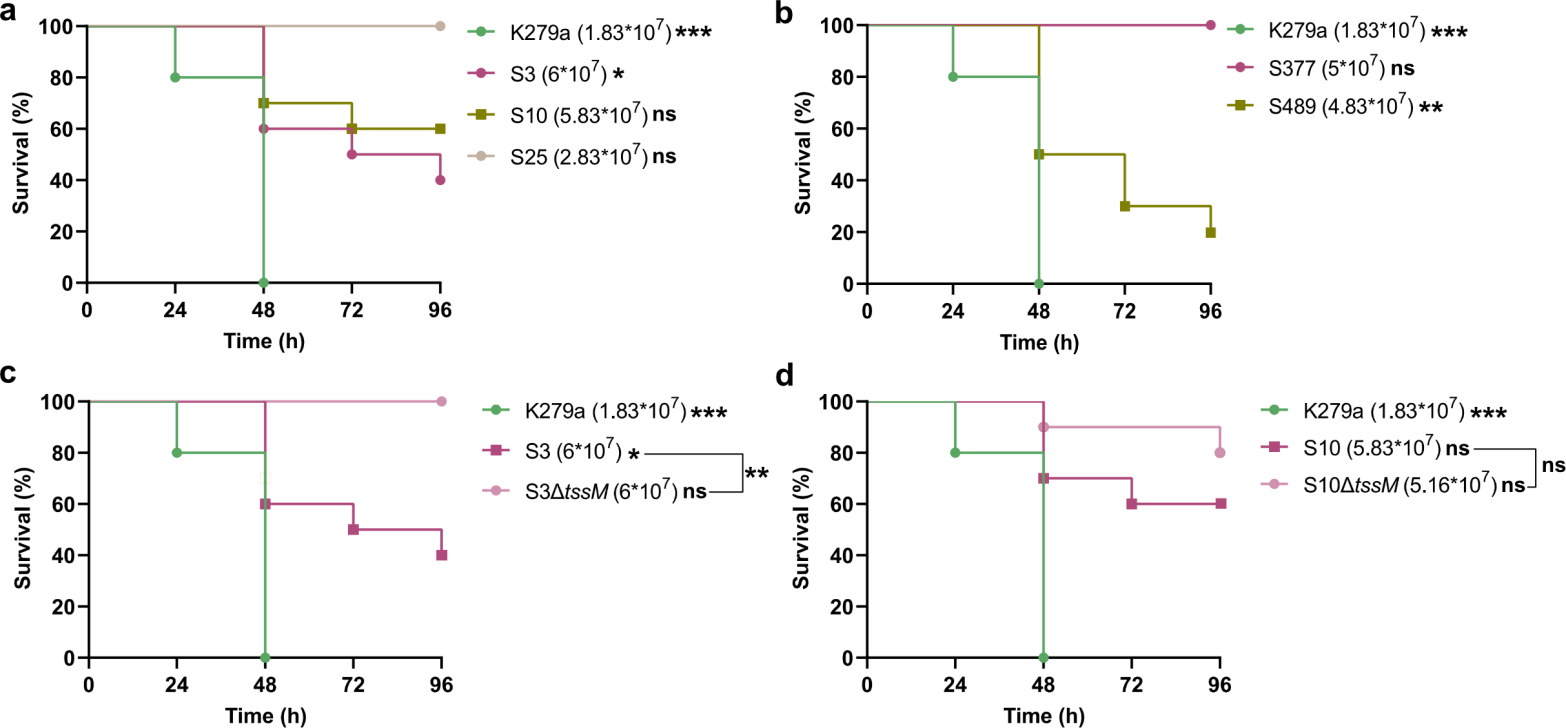
Virulence of *S. maltophilia* against *G. mellonella* larvae is T6SS-dependent in clinical isolates S3 and S10. Ten *G. mellonella* larvae were inoculated with roughly 10^7^ CFU of the respective *S. maltophilia* clinical (**a**; **c and d**) and environmental (**b**) strains. Larvae were incubated at 37°C for 96 h, and their mortality was scored daily. All experiments included a mortality positive control using strain K279a, as well as a negative control group of five larvae inoculated with PBS solution. Each survival curve was compared to the negative control (PBS), and the statistical significance is indicated next to each strain. Survival curves of larvae inoculated with S3 and S10 and their T6SS mutant counterparts were also compared, and the significance is shown next to the bracket (*, *P* < 0.05; **, *P* < 0.01; ***, *P* < 0.001; ns, not significant). Complete statistical analyses are provided in Table S7.

Next, we were interested in analyzing the virulence profile of our *S. maltophilia* isolates against the well-established model of *G. mellonella* larvae (81). Pilot experiments were performed in order to evaluate the best inoculum concentration to be used, and it was set to 10⁷ CFU. Reference clinical strain K279a was used as a control in all experiments, as this strain was able to kill 100% of *G. mellonella* larvae after 48 h of incubation at 37°C, using an inoculum of roughly 2 × 10^7^ (Fig. 6). Clinical isolates S3 and S10 were virulent, killing 40% and 60% of the tested larvae after 96 h, respectively (Fig. 6a). In contrast, S25 was not virulent to *G. mellonella*, with all larvae surviving 4 days after inoculation. Notably, the virulence of strains S3 and S10 against *G. mellonella* was considerably abrogated when T6SS mutants of these strains were used in the injections, with 100 and 80% of larvae surviving 96 h after injection with Δ*tssM* variants of strains S3 and S10, respectively (Fig. 6c and d).

In the case of environmental isolates, while all larvae survived 96 h after injection with strain S377, only around 20% of the population survived after injection with strain S489 (Fig. 6b). This result is strikingly similar to what was observed in our interbacterial competition experiments, where clinical strains S3 and S10 and environmental isolate S489 were able to outcompete *E. coli* much more effectively than the other tested isolates (Fig. 3a).

Furthermore, considering that environmental isolates would be expected to be less adapted to the human host, we tested their virulence when incubating the inoculated larvae at 28°C. As can be seen in Fig. S5a, while the S377 isolate was unable to kill any larvae at 37°C, only 30% of the population survived after 96 h of incubation at 28°C, demonstrating a virulent phenotype at this lower temperature. This increase in potency is comparable to what we observed in the interbacterial competition experiments as well, when this strain was able to kill around 50-fold more bacteria when experiments were performed at 30°C compared to 37°C (Fig. 3; Fig. S4a). In contrast, the virulence of strain S489 was attenuated at 28°C, with a larval mortality rate of 60% after 96 h, as compared to 100% at 72 h after injection when the incubation was performed at 37°C (Fig. S5b).

## DISCUSSION

*S. maltophilia* is an emerging pathogen of growing concern in hospital settings, primarily due to its high resistance to multiple antibiotics. However, this genetically diverse species complex also includes environmental lineages, with strains isolated from a wide range of sources, including soil, water, and sewage. Large-scale genomic studies suggest that the complex originated in the environment and that multiple events of pathogenic adaptation may have occurred during its evolution (4). *S. maltophilia* is frequently found in polymicrobial communities, both in clinical and environmental samples. Within these communities, bacteria must compete for limited resources and space, often employing various strategies to gain an advantage. Despite this, the mechanisms of interbacterial competition in *S. maltophilia* remain poorly understood. Initial studies indicate that some strains may possess an antibacterial T4SS (27–29), while others contain one or more T6SS clusters (30). However, these systems appear to be relatively rare across the phylogeny (30), presumably because the *S. maltophilia* complex is so genomically diverse (4).

In this study, we evaluated the distribution, functionality, and effector diversity of T6SS clusters in a set of *S. maltophilia* strains that have been recently isolated from patient samples (such as blood, oropharyngeal secretion, and sputum) as well as from diverse environmental sources (such as landfill and soil associated with different crops).

Our comparative and phylogenetic analyses showed that our seven sequenced Smc isolates were generally quite distinct from each other: three of them belong to three separate monophyletic groups (as defined by Gröschel et al. [4]), three belong to a fourth monophyletic group, and the last (S377) belongs to an uncharacterized lineage. Interestingly, there is complete consistency between the clades and the presence or absence of secretion systems (only i4 T6SS; i4 and i3 T6SS; and T4SS). This separation suggests that the ancestors of each of these clades already had the same secretion systems that we see in the present-day species; additional evidence for this hypothesis could, in principle, be obtained by a phylogenetic analysis based only on T4SS and T6SS gene sequences.

From our collection of 29 clinical and 13 environmental *S. maltophilia* isolates with confirmed identities, three clinical (10.3%) and two environmental (15.4%) strains were found to carry T6SS clusters. A recent study analyzing 835 *Stenotrophomonas* genomes from NCBI reported that 8.3% (64 strains) contained at least one T6SS cluster (69). Another study, focused specifically on the Smc, analyzed approximately 1,000 genomes from GenBank and RefSeq and found a slightly lower prevalence of 6.1% (30). Despite the smaller size of our data set, our results support the notion that T6SS clusters are generally rare in *S. maltophilia*.

T6SS clusters can be classified into phylogenetic groups based on the amino acid sequence of TssC, a structural sheath protein (66). In our data set, all five T6SS-positive isolates carried an i4-clade cluster, inserted at the same genomic location and with a conserved backbone. The i4 clade was also the most abundant among T6SS clusters identified in *Stenotrophomonas*, representing 47 of 64 genomes (69), and was similarly prevalent in the Smc (30). Interestingly, one clinical strain in our collection, S25, harbored an additional i3-type cluster, closely resembling the anti-eukaryotic T6SS from *X. citri* (67). Strains with both i3 and i4 clusters have also been described in *Stenotrophomonas*, including a clade of nine closely related Smc strains (69). Similarly, in a data set of 1,000 Smc genomes, 17 of the 21 i3-positive strains also carried an i4 cluster (30). Our phylogenetic analysis showed that all strains closely related to S25 also harbor both clusters, suggesting that this configuration is conserved and potentially advantageous in this lineage. The presence of specific T6SS clusters in Smc appears to be closely correlated with phylogenetic relatedness, consistent with previous findings (5). Our data show a similar trend for T4SS clusters, reinforcing the idea that both systems are lineage-specific.

Given that *X. citri*’s i3 cluster functions as an anti-eukaryotic weapon, conferring resistance against amoeba predators (67), the presence of a second T6SS cluster in this lineage may reflect a “division of labor,” where each system targets distinct competitors or functions under different conditions (e.g., for metal acquisition). This functional specialization is observed in *Burkholderia thailandensis*, which encodes five T6SS clusters: T6SS-1 (interbacterial competition), T6SS-4 (manganese scavenging), and T6SS-5 (virulence via cell-to-cell spread) (24, 82–84). Likewise, *P. aeruginosa* carries multiple T6SS clusters—H1 (antibacterial) and H2/H3 (versatile roles including anti-eukaryotic, antibacterial, and exploitative activities). These three systems are found in 96.6% of all 2,000 *P. aeruginosa* public genomes, highlighting their centrality in this species’ biology, while a fourth system (H4-T6SS) is more sporadically encountered (76).

In our interbacterial competition assays, three strains (S3 and S10, clinical; S489, environmental) killed *E. coli* more effectively than S25 and S377. These three strains also showed increased virulence against *G. mellonella* larvae. Most importantly, for S3 and S10, we confirmed that both phenotypes were T6SS-dependent using *tssM* mutants; however, the lack of genetic complementation of the *tssM* mutants represents a limitation of this study. Notably, the three strains are all part of the Sgn4 monophyletic clade (4), despite originating from different environments. They share a similar T6SS effector repertoire—except for a VgrG1-associated effector in S489, which remains unconfirmed—suggesting that either their T6SS is more active, or their effectors are particularly potent. Regarding the virulence of *S. maltophilia* against *G. mellonella*, it is unclear whether the T6SS would directly target host cells or act indirectly through interactions with the host microbiota or immune system. *G. mellonella*’s microbiota is dominated by gram-positive bacteria, particularly *Enterococcus* (85), and the T6SS is generally less effective against such organisms. Thus, it is plausible that these *S. maltophilia* isolates deploy some T6SS effectors directly against host cells. The main T6SS cluster in these strains encodes two predicted DUF2235 phospholipases, a pore-forming toxin, and a peptidoglycan-degrading enzyme. While these can mediate interbacterial competition, some (e.g., phospholipases, pore-forming toxins) may also have anti-eukaryotic activity. For instance, *P. aeruginosa*’s phospholipase PldB (H3-T6SS) promotes both antibacterial action and host cell invasion (36, 73, 86). Similarly, the VgrG1-associated pore-forming toxin in S3 and S10 has a VasX-like domain, known to mediate both antibacterial and anti-amoebic functions in *V. cholerae* (87, 88). Further research is needed to determine the exact mechanisms and roles of these effectors.

A recent study tested 13 Smc isolates from cystic fibrosis patients against *E. coli*, *P. aeruginosa*, and *S. aureus*. While nine isolates killed *E. coli* below detection limits, effects on the other species were minimal (89). Over half the isolates in that study belonged to the Sm6 lineage, consistent with its overrepresentation in genome databases (4). Conversely, Sm6 was represented by a single environmental isolate in our study (S370), which, like others in this lineage, carries a T4SS. In the study by Crisan et al. (89), only two isolates carried T6SS clusters, including strain CCV119, a member of the Sgn4 clade—like S3, S10, and S489. Notably, CCV119 completely eliminated *E. coli* and showed moderate activity against *S. aureus*.

Differences in antibacterial activity between strains, a trend observed in our study and also by Crisan et al. (89), can have multiple explanations. The T6SS is generally tightly regulated by signals perceived by the bacteria, such as osmolarity, oxygen, pH, and temperature, or the presence of biotic compounds such as chitin, mucin, antibiotics, and quorum-sensing molecules (90). We observed mild temperature effects: incubating competition plates at 30°C slightly enhanced the killing ability of S25 and S377 but reduced it in S3, S10, and S489. These effects were independent of strain origin, unlike *V. cholerae*, where low temperatures enhance T6SS expression in pandemic strains but not in non-pandemic ones (91). Moreover, although all our Smc isolates carry the same i4-type T6SS cluster, subtle regulatory differences may influence expression—similar to how a single SNP affects T6SS activity in *V. cholerae* strains (92, 93). Additionally, some isolates may only activate their T6SS defensively, responding to attacks from competitors. These “counterattack” systems require specific triggers like membrane damage or antibiotic exposure (90) and are well-characterized in *P. aeruginosa* (94–96).

We did not test the functionality of the i3-type T6SS cluster in S25 in this study. This cluster is nearly identical to the anti-predation system of *X. citri* (67), with variation observed only among genes encoding hypothetical proteins. The T6SS cluster in S25 includes two open reading frames (ORFs) between *clpV* and *paar*, occupying the same region as *X. citri*’s inter-cluster region. These ORFs are non-overlapping and separated by 63 bp, making them unlikely to encode typical effector/immunity pairs. Future research will investigate the functionality of this cluster and explore the conditions that promote expression of the i4 cluster in our Smc isolates.

Our findings highlight the low overall prevalence but notable lineage-specific conservation of T6SS clusters within *S. maltophilia*. The co-occurrence of i3 and i4 T6SS clusters in certain lineages suggests potential functional specialization, possibly enabling dual targeting of bacterial and eukaryotic competitors. Interbacterial competition and virulence assays revealed that Sgn4 strains S3, S10, and S489 exhibit enhanced antibacterial activity and increased pathogenicity in *G. mellonella*, in a T6SS-dependent manner. These results support the idea that both T6SS presence and effector composition contribute to strain-specific ecological strategies and virulence potential. Furthermore, the apparent regulation of T6SS activity by environmental cues such as temperature underscores the importance of context-dependent expression in determining competitive fitness. Future work should explore the regulation and function of these clusters in Smc, as well as the interplay between T6SS and T4SS systems across diverse *S. maltophilia* lineages.

## Data Availability

This Whole Genome Shotgun project has been deposited at DDBJ/ENA/GenBank under the accession PRJNA1232787. Genome accession numbers are provided in Table S3.

## SUPPLEMENTAL MATERIAL

File S1 spectrum.03411-25-s0001.docx Figures S1 to S5 and Tables S1 to S4.

File S2 spectrum.03411-25-s0002.xlsx Tables S5 to S7.

ASM does not own the copyrights to Supplemental Material that may be linked to, or accessed through, an article. The authors have granted ASM a non-exclusive, world-wide license to publish the Supplemental Material files. Please contact the corresponding author directly for reuse.
