# Supplementary material for: Type VI-secretion system-mediated competition and virulence in clinical and environmental Stenotrophomonas maltophilia
Source: Microbiol Spectr. 2026 Feb 27;14(4):e03411-25. doi: 10.1128/spectrum.03411-25 (PMC13055248; doi:10.1128/spectrum.03411-25)
Supplement: File S1 — Figures S1 to S5 and Tables S1 to S4. [file spectrum.03411-25-s0001.docx]

**Supplementary Information**

**Type VI-Secretion System Mediated Competition and Virulence in Clinical and Environmental *Stenotrophomonas maltophilia***

**Authors:** Natália Carolina Drebes Dörr^1^; Bárbara Bortolozzo Ribeiro^1^; Arthur Henrique Barrios Solano^2^; Isabella Carolina Rodrigues^1^; Francine Coelho Rosa^1^; Natalli Jennifer Tancredo de Oliveira^1^; Eliana Guedes Stehling^3^; Herrison Fontana^4^; Marcelo Brocchi^1^; Rodrigo S. Galhardo^4^; João Carlos Setubal^2^; Cristina E. Alvarez-Martinez^1*^

**Supplementary figures**


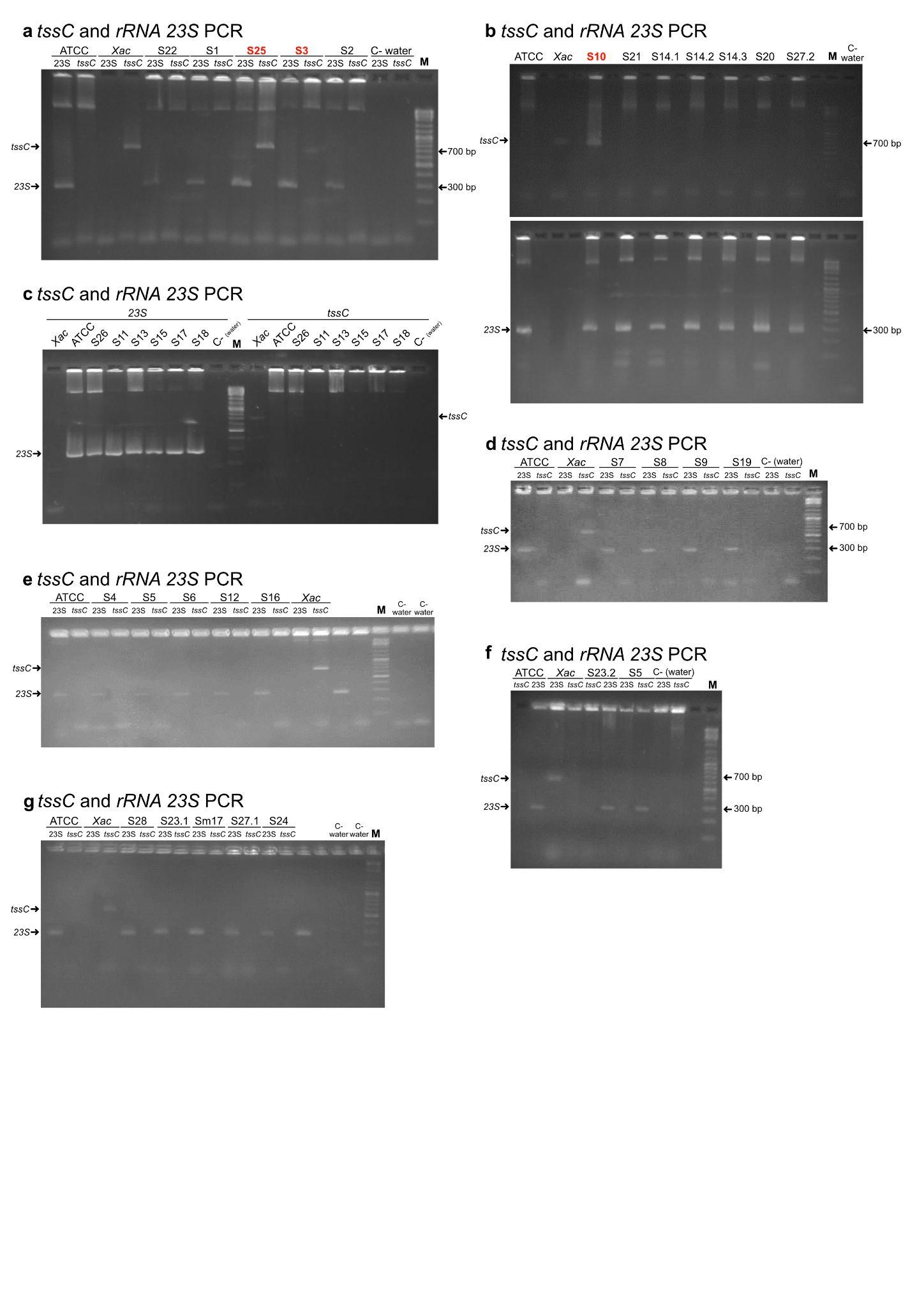


**Supplementary Figure 1. Detection of T6SS-positive *S. maltophilia* clinical isolates by *tssC* degenerate PCR.** Presence of a *tssC* gene (indicative of a possible T6SS cluster) was tested in *S. maltophilia* clinical isolates by colony PCR amplification using degenerate primers (described in Suppl. Table 2). A *tssC* from clades i3 and i4 should result in a 713-bp fragment. Amplification of the 278-bp *23S rRNA* fragment was performed in parallel, to attest colony DNA quality. A positive control for *tssC* amplification (*X. citri*’s genomic DNA) and for *23S* amplification *(S. maltophilia* ATCC13637’s genomic DNA) were included, as well as a negative control using water.


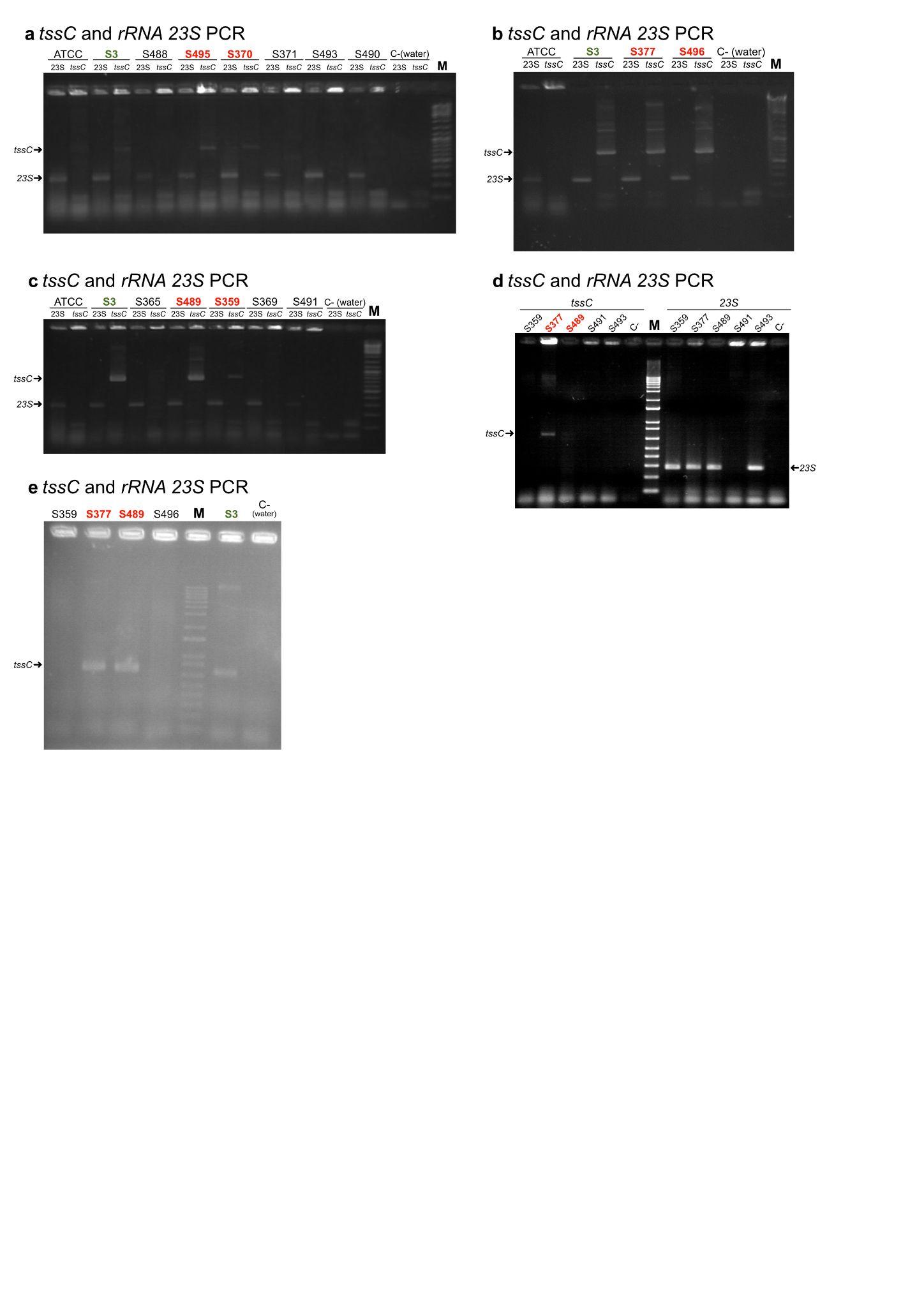


**Supplementary Figure 2. Detection of T6SS-positive *S. maltophilia* environmental isolates by *tssC* degenerate PCR.** Presence of a *tssC* gene (indicative of a possible T6SS cluster) was checked in *S. maltophilia* environmental isolates by colony PCR amplification using degenerate primers (described in Suppl. Table 2). A *tssC* from clades i3 and i4 should result in a 713-bp fragment. Amplification of the 278-bp *23S rRNA* fragment was performed in parallel, to attest colony DNA quality. A positive control for *tssC* amplification (genomic DNA of *S. maltophilia* S3 strain) and for *23S* amplification *(S. maltophilia* ATCC13637’s genomic DNA) were included, as well as a negative control using water. Initial screenings **(a-c)** pointed to six T6SS-positive isolates (S359, S370, S377, S496, S489 and S495), but only two isolates (S377 and S489) were confirmed by additional PCR screening and whole-genome sequencing **(d-e)**.


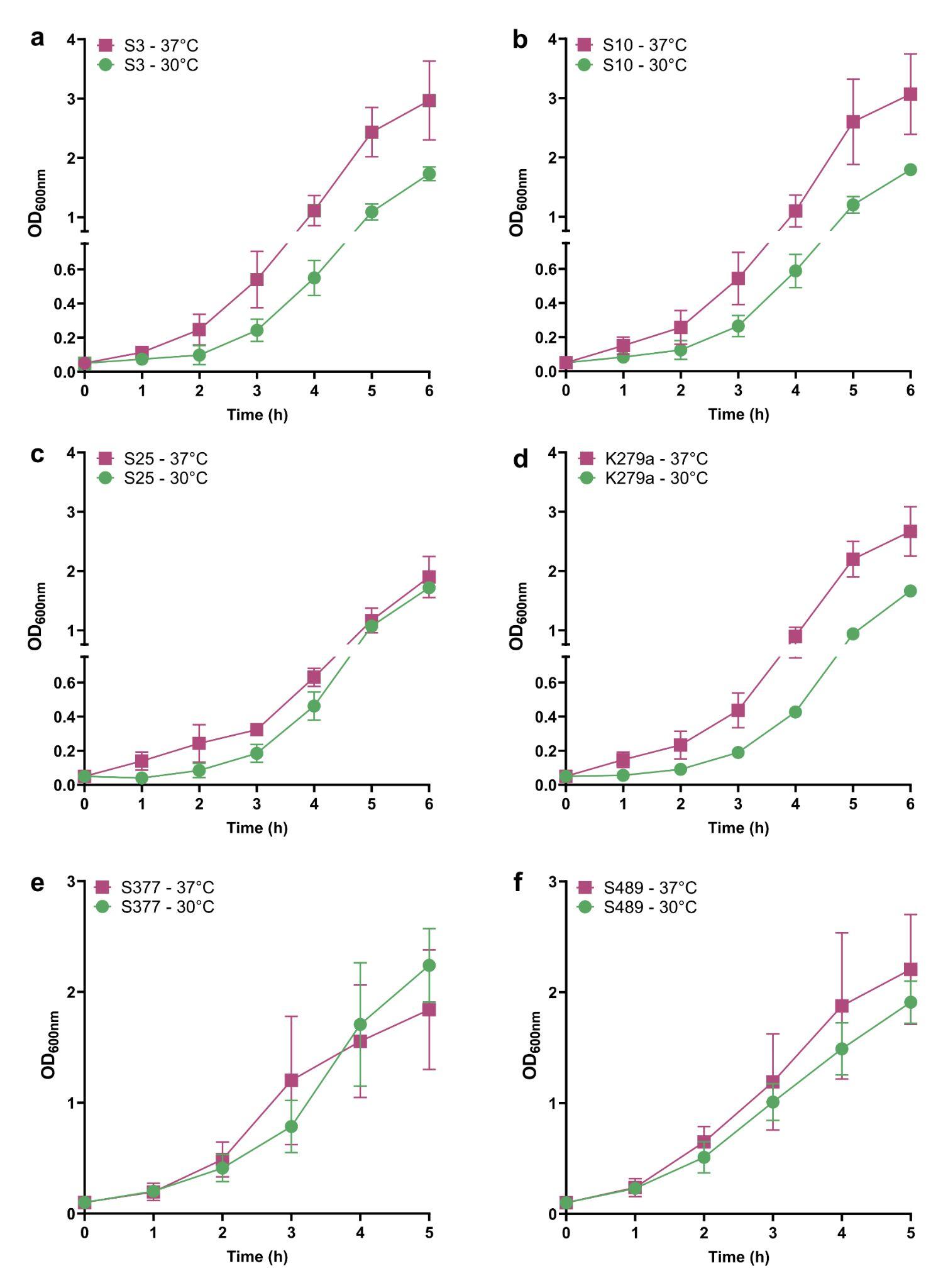


**Supplementary Figure 3. Growth curves of *S. maltophilia* strains at 30°C and 37°C.** Growth of *S. maltophilia* strains in TSB medium was inferred by absorbance (OD_600_) every hour, after initial back-dilution of overnight cultures to an OD_600_ of 0.05 (clinical strains; **a-d**) or 0.1 (environmental strains; **e-f**).

**
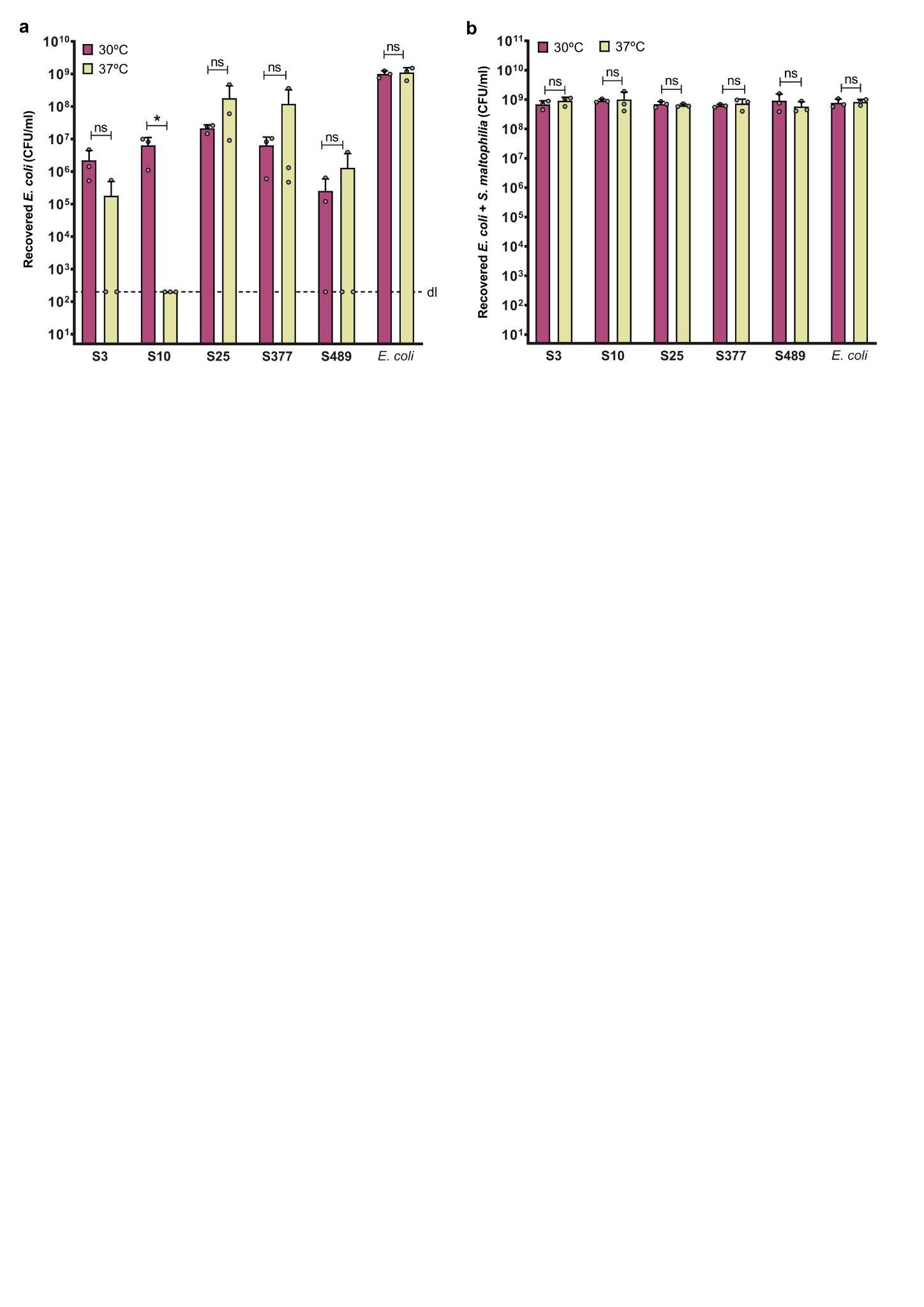
**

**Supplementary Figure 4. Recovery of prey and predator bacteria after competition experiments at 30°C or 37°C.** Bacterial killing assays using *E. coli* as prey. *S. maltophilia* strains and *E. coli* prey were mixed at a 20:1 ratio, spotted onto LB agar plates and incubated for 20 h at 30 or 37°C. Prey recovery alone is shown in **(a)** (results from Fig. 3a and Fig. 3b are combined). The numbers of prey and predator (*E. coli* and *S. maltophilia*; CFUs in LB without antibiotics) recovered after competition are depicted in **(b)**. Bar plots in both panels represent the average of three independent biological replicates (±SD), which are indicated by small gray circles. dl, detection limit. Statistical significance is shown using two-way ANOVA followed by Sidák’s multiple comparisons test. Numbers of bacteria recovered at 30°C and 37°C were compared for each strain tested. *, *P* < 0.05; n.s., not significant.


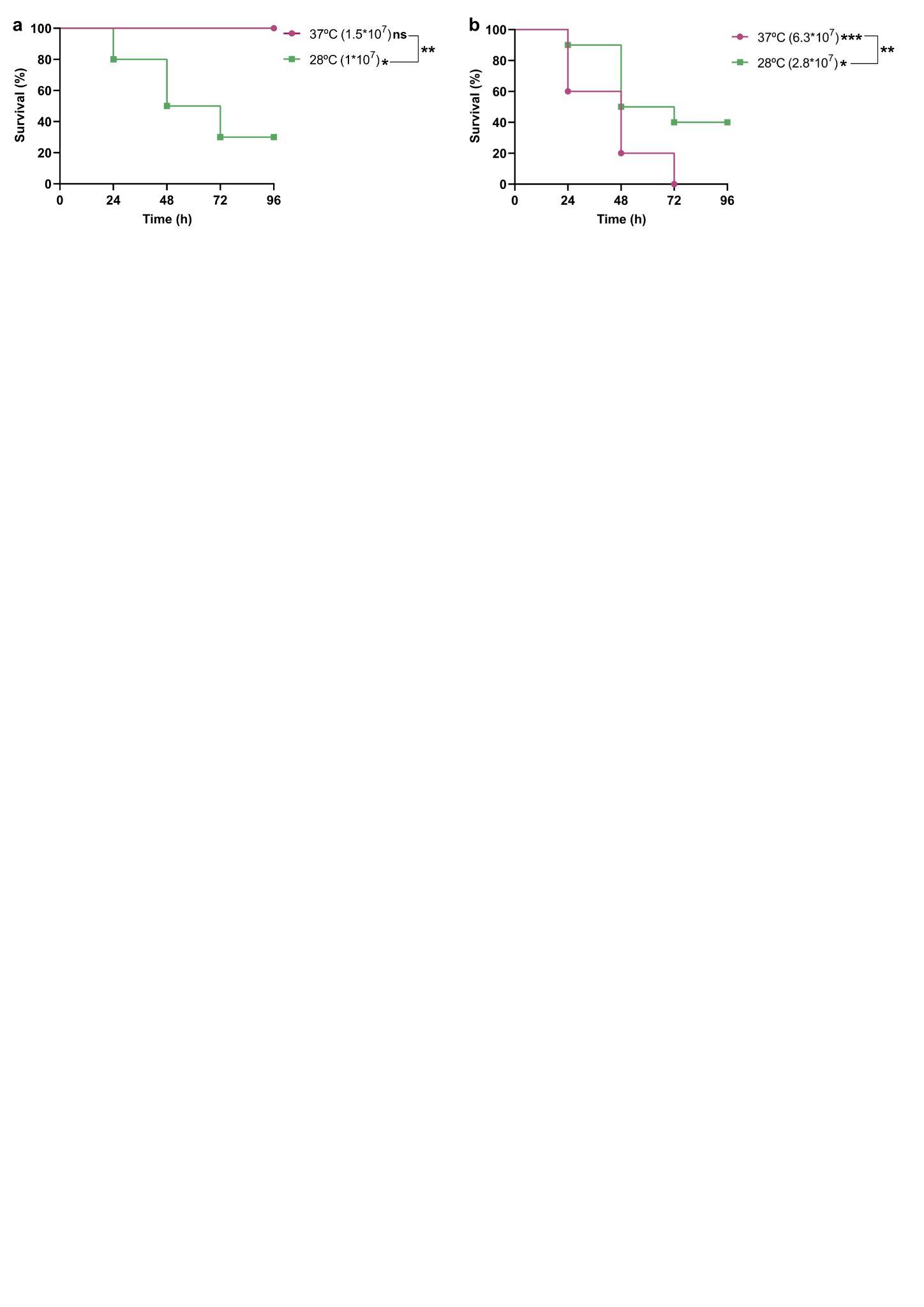


**Supplementary Figure 5. Temperature-dependency in virulence of *S. maltophilia* environmental isolates against *G. mellonella* larvae.** Ten *G. mellonella* larvae were inoculated with roughly 10^7^ CFU of environmental *S. maltophilia* isolates S377 **(a)** and S489 **(b)**, which had been grown at 28°C or 37°C. Inoculated larvae were incubated at 28°C or 37°C for 96 hours, and their mortality was scored daily. All experiments included a mortality positive control using strain K279a (not shown), as well as a negative control group of 5 larvae inoculated with PBS solution. Each survival curve was compared to the negative control (PBS), and the statistical significance is indicated next to each temperature. Both temperature survival curves using each strain were also compared, and the significance is shown next to the bracket ( *, *P* < 0.05; **, *P* < 0.01; ***, *P* < 0.001; n.s., not significant). Complete statistical analyses are provided in Supplementary table 7.

**Supplementary tables**

**Supplementary Table 1.** Additional microorganisms used in this study.

| **Strain** | **Description** | **Reference** |
| --- | --- | --- |
|  |  |  |
| *Escherichia coli* DH5ɑ | F- endA1 glnV44 thi- recA1 relA1 gyrA96 deoR nupG purB20 φ80dla cZΔM15 Δ(lacZYA - argF) U169, hsdR17(rK-mK+), λ- | [(1)](https://www.zotero.org/google-docs/?yhY0mz) |
| *E. coli* MG1655 *argA*::Tn10-Tet^R^ | K12; λ- F- rph-1 rfb-50 ilvG-; Tn10-Tet^R^ insertion in *argA* | Laboratory collection, gift from Prof. Beny Spira (ICB/USP) |
| *E. coli* S17-λpir | Tpr Smr recA thi pro hsdR2M1 RP4:2-Tc:Mu:Kmr Tn7 (λpir); Str^R^ | [(2)](https://www.zotero.org/google-docs/?IKOctT) |

**Supplementary Table 2.** Primers used in this study.

| **Primer** | **Sequence** | **Application** |
| --- | --- | --- |
|  |  |  |
| ggpSG3i | CAACGGCGTGATCCACAT | *ggpS* |
| ggpSG5i | GATGCTGCATGTGTTCTG |  |
| smeD3 | CCAAGAGCCTTTCCGTCAT | *smeD* |
| smeD5 | TCTCGGACTTCAGCGTGAC |  |
| EBS15-F | GCTGGATTGGTTCTAGGAAAACGC | *23S rRNA* |
| EBS16-R | ACGCAGTCACTCCTTGCG |  |
| Fo-tssC Adeg* | TGGCGBGGBCTGCAYTAYCTG | *tssC* - clades 3 and 4 |
| Re-tssC Adeg* | GCSACYTCVGTSGGGCACTT |  |
| Fo-tssM-1deg | TGGCCSCARGTGCARAASAAG | *tssM* clade 4 |
| Re-tssM-1deg | CTTGAASGGRTCRTASACCTG |  |
| F1_for_tssM_SM03_XbaI | CCTCTAGACGTCAGGACGGCATCGACAC | construction of pEX18Tc plasmid for *tssM* deletion |
| F1_rev_tssM_SM03 | GGCAGGGGTCAGGTAGTAGCTGAAATTGCTCAAACTCATGC |  |
| F2_for_tssM_SM03 | CAGCTACTACCTGACCCCTGCCGCGCCC |  |
| F2_rev_tssM_SM03_HindIII | CCAAGCTTCAGCTTGCGTGCGGTGGC |  |
| tssM_SM03_chk_up | CGTTGGCGCAGCTGGCACT | chromosomal check of *tssM* deletion |
| tssM_SM03_chk_down | GTCGGCCGCACGGACGAAATC |  |

**For degenerate primers, B = C, G or T; Y = C or T; S = C or G; H = A, C or T; V = A, C or G; R = A or G; M = A or C; Y = C or T*

**Supplementary Table 3.** NCBI accession numbers of genomes deposited in Whole Genome Shotgun project PRJNA1232787.

| **Strain** | **BioSample** | **Accession** | **Organism (NCBI)** |
| --- | --- | --- | --- |
|  |  |  |  |
| S3 | SAMN47245849 | JBMDMA000000000 | *S. maltophilia* S3 |
| S10 | SAMN47245850 | JBMDLZ000000000 | *S. maltophilia* S10 |
| S25 | SAMN47245855 | JBMDLY000000000 | *S. riyadhensis* S25 |
| S370 | SAMN47245851 | JBMDLX000000000 | *S. maltophilia* S370 |
| S377 | SAMN47245852 | JBMDLW000000000 | *S. maltophilia* S377 |
| S489 | SAMN47245853 | JBMDLV000000000 | *S. maltophilia* S489 |
| S495 | SAMN47245854 | JBMDLU000000000 | *S. geniculata* S495 |

**Supplementary Table 4.** Plasmids used in this study.

| **Plasmid** | **Description** | **Reference** |
| --- | --- | --- |
|  |  |  |
| pEXT18Tc | ColE1 replicon, *oriT*, *tet* (Tet^R^), *sacB*, suicide vector used to perform allelic exchange in *S. maltophilia*. Tet^R^ | [(3)](https://www.zotero.org/google-docs/?uMHehm) |
| pEXT18Tc-*ΔtssM* | For *in frame* deletion of *tssM*. Tet^R^ | This study. |

**References**

[1.](https://www.zotero.org/google-docs/?0PvpAY)  [Hanahan D. 1983. Studies on transformation of Escherichia coli with plasmids. J Mol Biol 166:557–580.](https://www.zotero.org/google-docs/?0PvpAY)

[2.](https://www.zotero.org/google-docs/?0PvpAY)  [Simon R, Priefer U, Puhler A. 1983. A broad host range mobilization system for in vivo genetic engineering: transposon mutagenesis in Gram negative bacteria. Nat Biotechnol 1:784–791.](https://www.zotero.org/google-docs/?0PvpAY)

[3.](https://www.zotero.org/google-docs/?0PvpAY)  [Hoang TT, Karkhoff-Schweizer RR, Kutchma AJ, Schweizer HP. 1998. A broad-host-range Flp-*FRT* recombination system for site-specific excision of chromosomally-located DNA sequences: application for isolation of unmarked *Pseudomonas aeruginosa* mutants. Gene 212:77–86.](https://www.zotero.org/google-docs/?0PvpAY)
